# Reaction Kinetics using a Chemputable Framework for Data Collection and Analysis

**DOI:** 10.1002/anie.202315207

**Published:** 2024-01-24

**Authors:** Bartosz M. Matysiak, Dean Thomas, Leroy Cronin

**Affiliations:** ^1^ School of Chemistry University of Glasgow Glasgow G12 8QQ UK

**Keywords:** Automation, Chemputation, Data Acquisition, Kinetics, Robotics

## Abstract

Automated chemistry platforms have been widely explored, but many focus on fixed tasks for chemical synthesis or analysis. However, a typical synthetic chemistry workflow utilizes both, such as kinetic measurements for reaction development and optimization. Due to their repetitive and time‐consuming nature, kinetic measurements are often omitted, which limits the mechanistic investigation of reactions. Herein, we present a “Chemputer” platform with on‐line analytics (UV/Vis, NMR) which automates routine kinetic measurements. The system's capabilities are showcased by exploring an inverse electron‐demand Diels–Alder using initial rate measurements, a metal complexation using variable time normalization analysis (VTNA), and formation of a series of tosylamide derivatives using Hammett analysis. Over 60 individual experiments are presented which required minimal intervention, highlighting the significant time savings of automation. Owing to the modular design of the platform, which facilitates rapid integration of commercial analytical tools, our approach is widely accessible and adjustable to the reaction under investigation. The platform is operated using the chemical programming language, XDL, hence experimental procedures and results are stored in a precise, computer‐readable format. We propose that widespread adoption of this reporting protocol in the chemical community could build a database of validated kinetic data beneficial for Machine Learning.

## Graphical abstract

The ChemPU platform streamlines chemistry by automating kinetic measurements, bridging synthesis and analysis. It's versatile, adaptable, and facilitates precise data encoding of reactions via the Chemical Description Language (XDL). This innovation has the potential to enhance efficiency and create a valuable database of kinetic data for broader applications.

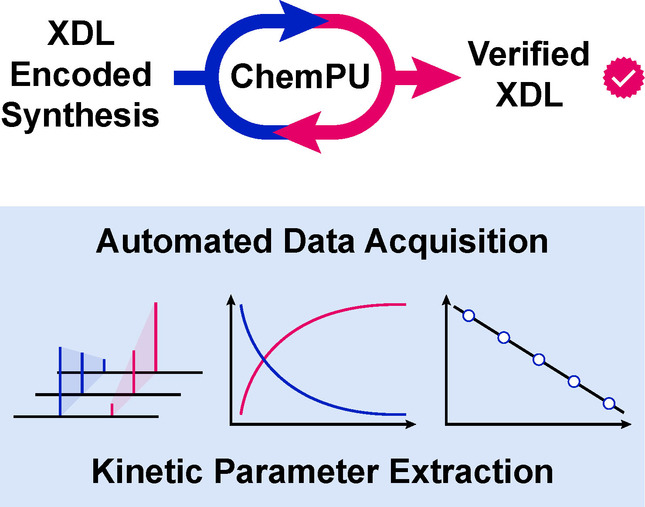

## Introduction

Reaction monitoring is a fundamental necessity for chemists to successfully understand the examined process in more detail, enabling optimization, mechanistic investigation and scale‐up.[^1^, ^2^, ^3^, ^4^, ^5^, ^6^] Over the years, many methodologies were established to probe the kinetic behavior of chemical reactions. Traditional methods focus on initial rate measurements, which determine various kinetic parameters using only the early linear portion of a kinetic trace. More recently, new, graphical kinetic methodologies are being established, such as Reaction Progress Kinetic Analysis (RPKA)^[1]^ and Variable Time Normalization Analysis (VTNA).^[3]^ Compared to traditional initial rate measurements, these methods operate at or near synthetic conditions, making the results significantly more representative of the reaction mechanism. For that reason, these approaches are being widely adopted, especially in catalysis community, as the catalytic cycles can oftentimes be drastically affected by the concentration regime under study.

In some studies, chemists typically focus on obtaining the product of their reaction and refer to kinetic data only in cases where troubleshooting is required. This is understandable given the time‐consuming nature of kinetic analysis, however valuable information is often lost due to such a product‐driven approach. We think the general understanding of chemical reactivity would be transformed by the incorporation of kinetic data acquisition into the standard chemical operating procedure. Often, we are able to obtain products without true understanding of the mechanism of the reaction, or the mechanism for given substrates might deviate from the established general one, despite giving the same products. This means the acquisition of kinetic (hence mechanistic) data as part of a standard protocol would help our understanding reactivity patterns. In this way we could enter a new paradigm of chemistry where obtaining the chemicals of interest and the knowledge of fundamental reactivity patterns are coupled together. For this to be possible, kinetic measurements need to be performed without an excess burden to the chemist, which is possible with automation.^[7]^

Automation has long played an important role in chemical and pharmaceutical industry,^[8]^ however it is now becoming present in the academic setting as well, thanks to the increasing economic accessibility.^[9]^ Typically, robotic platforms are limited to the execution of routine tasks, *vide* autosamplers, sample preparation stations or flash chromatography systems. Performing synthesis in an automated fashion remains almost exclusively a bespoke technology,^[10]^ with flow systems constituting a major share of examples,^[11]^ although alternate approaches are known.^[12]^ The requirement to develop new hardware (and non‐standardized software) for each experiment further restricts the successful integration of automation in a chemist's toolkit.

Over recent years, we have developed the Chemputer platform, a universal synthesis machine operated by Chemical Description Language (XDL), capable of performing the majority of organic synthetic procedures reported in the literature.[^13^, ^14^, ^15^, ^16^] Owing to its modular character, the automated platform can be expanded with hardware units required for the particular reaction of interest. Furthermore, it allows for inclusion of process analytical technology (PAT) tools,^[17]^ which we have previously used to find and optimize new chemical transformations.[^13^, ^18^, ^19^, ^20^]

In each of those studies, different in‐line, on‐line and at‐line analytical techniques were used, including nuclear magnetic resonance (NMR), mass spectrometry (MS), UV/Vis and Raman spectroscopy and high‐pressure liquid chromatography (HPLC). Additionally, others have shown the use of fluorescence spectroscopy and gas chromatography (GC), both in flow and in batch systems.[^7^, ^17^, ^21^, ^22^, ^23^, ^24^, ^25^] In this work, we apply UV/Vis^[19]^ and NMR spectroscopy,^[26]^ connected to a standard Chemputer platform,[^13^, ^14^, ^15^, ^16^] to perform kinetic measurements on series of different reactions. Additionally, we run those measurements as part of a standard synthetic procedure to showcase the potential of seamlessly integrating kinetic data acquisition in automated synthetic platforms.

## Results and Discussion

**Figure 1:**
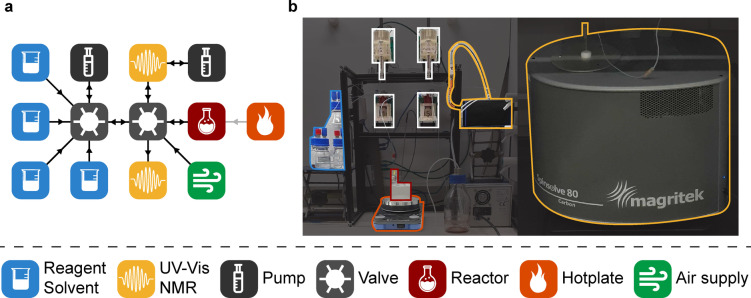
[a] Graph scheme of the Chemputer setup used in this study; [b] Photograph of the Chemputer platform, including a Magritek Spinsolve 80 benchtop NMR spectrometer and an Avantes Avaspec‐DUAL 4096 UV/Vis spectrophotometer (Ocean Optics DH‐2000 light source and FIA‐Z‐SMA 905 flow cell).

In our study, we utilized the standardized Chemputer hardware, composed of a series of syringe pumps and six‐way selection valves, operated by the Chemical Description Language (XDL) (Figure 1).^[**15**]^ The setup was expanded with the analytical modules as required per reaction studied (See Supporting Information, Section 1). The analytical modules were controlled using a previously reported in‐house developed Python package ‐ AnalyticalLabware,^[27]^ that permits the acquisition and analysis of spectral data. This is achieved by utilizing and unifying connection interfaces provided by manufacturers for their respective instruments. The package currently gives access to a number of different PAT tools, including UV/Vis, near IR (NIR), Raman and NMR spectrometers, as well as HPLC‐DAD, but it also allows for easy integration of further modules. The package is part of our greater Chemputer workflow, as the analytical devices are represented in the graph via respective nodes with corresponding connection parameters, which can be addressed via XDL steps, specifying the sampling routine and data acquisition parameters. All the data acquired via this process is stored in open‐access formats, permitting facile integration with databases, which is of uttermost importance for use the of machine learning in chemistry.

Our initial investigations focused on UV/Vis spectrophotometry as the PAT tool. This form of reaction monitoring is particularly suited to reactions where a chromophore is generated, destroyed or its structure substantially changed. Furthermore, this analysis is popular due to the ease of operation and low‐cost of the commercial apparatuses (or even the numerous open‐source implementations).

**Figure 2:**
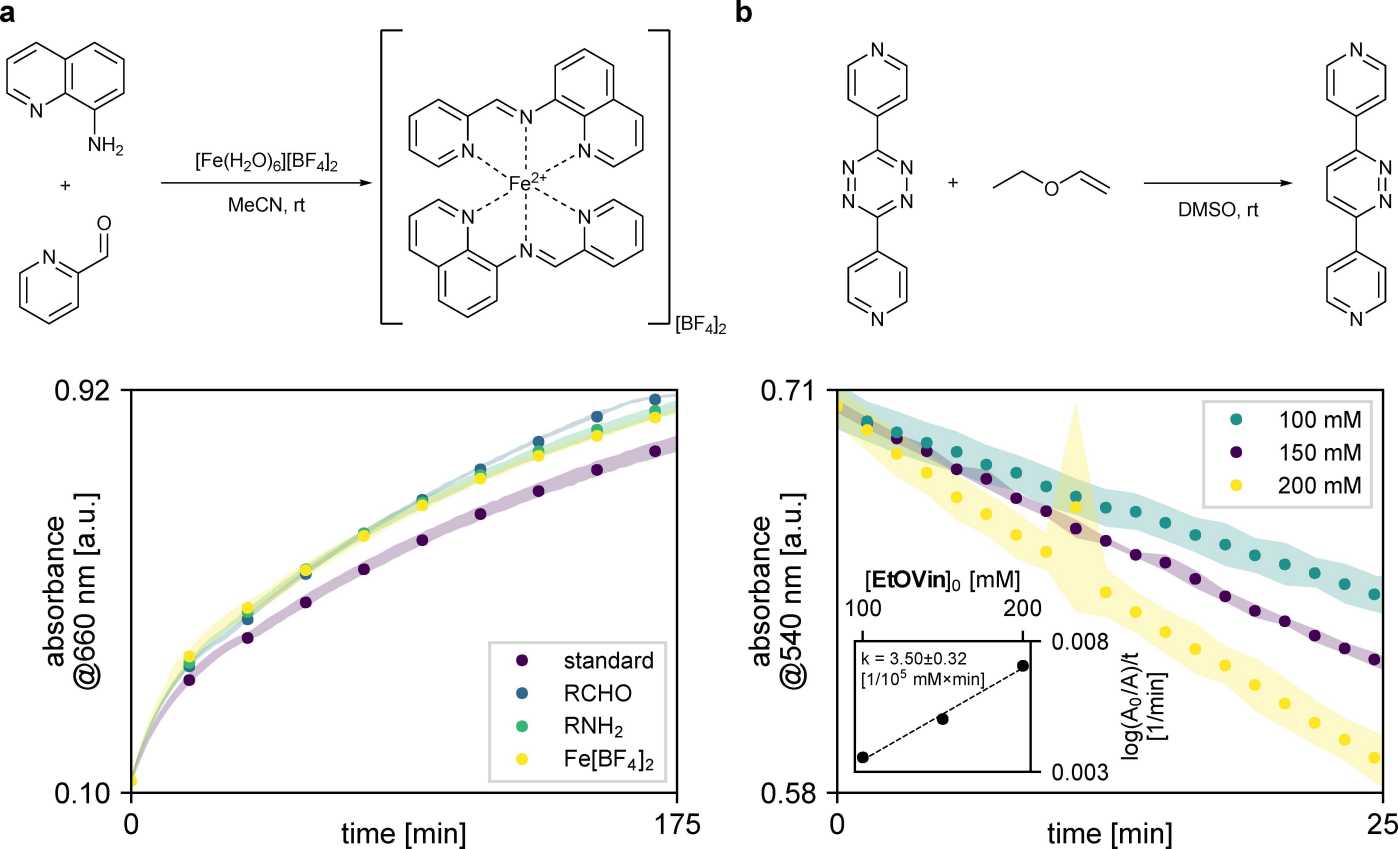
Results of the analysis conducted using UV/Vis spectroscopy as the process analytical technology (PAT). [a] Iron complex formation followed by visual kinetic analysis using different reagents in excess as compared to the standard reaction conditions; [b] Inverse electron demand Diels–Alder reaction of a tetrazine derivative followed by initial rate measurement under pseudo‐first order conditions.

To demonstrate the feasibility of our approach, we chose two reactions representing vastly different classes. The first reaction examined the formation of a transition metal complex between iron(II) ions and in situ generated imine ligands (Figure 2a).^[28]^ Transition metal compounds are often coloured due to the presence of partially filled d‐orbitals in the metal atoms, allowing for d‐d transitions, the energy of which typically falls within the visible light range. As the absorbance wavelengths can be heavily dependent on the coordination sphere, UV/Vis permits for the facile monitoring of the reaction progression. To analyse the reaction, we opted for visual kinetic analysis. For that purpose, we monitored a standard experiment, in which one equivalent of iron(II) tetrafluoroborate hexahydrate was mixed with two equivalents of 8‐aminoquinoline and two equivalents of 2‐formylpyridine in acetonitrile. The standard experiment was followed by three additional experiments, in which one of the reagents was added in excess (1.2 equivalents compared to the standard experiment). In all the experiments, the reagents were added consecutively to the reactor, after which the reaction was run for *ca*. three hours, with the measurements taken every 112 seconds. Comparing the kinetic traces obtained in these experiments, all reagents showed a rate increase compared to the standard experiment. The reaction is approximately first order with respect to 2‐formylpyridine, and 8‐aminoquinoline and further studies are required to elucidate the precise order of the iron(II) complex. The reaction Scheme presented does not do justice to the complexity of reaction network underneath, and more thorough studies would be required to fully grasp the mechanism beyond it (See Supporting Information, Section 4.1 for exemplar data and underlying assumptions).

The second reaction was an inverse electron demand Diels–Alder (IEDDA) reaction of a 1,2,4,5‐tetrazine derivative (Figure 2b), a transformation of importance in chemical biology thanks to its bio‐orthogonality. The transformation is known to be first order in both the diene and dienophile as it is a prime example of a pericyclic reaction.^[29]^ Building on that, we have decided to utilize initial rate measurements under pseudo‐first order conditions to determine the rate constant. For that purpose, we conducted three experiments, in which 1.5 mM tetrazine derivative was treated with 100/150/200 mM ethyl vinyl ether in dimethyl sulfoxide, respectively. The reactions were followed for 20 datapoints with the measurements taken every 82 seconds. The rate constant for this reaction was determined as 3.50±0.32×10^−5^ mM^−1^ min^−1^.

Subsequent investigations focused on proton NMR as the PAT tool. In recent years, increased economic accessibility of low‐field bench‐top apparatus made their use more feasible. The obvious advantage of proton NMR compared to other analytical techniques is that the signals can be observed for all compounds containing hydrogen atoms, making theoretically every organic reaction tractable (as long as a diagnostic region can be defined, and timescale of the reaction is long enough). In addition, the signals observed are directly proportional to the concentrations of analytes, which eliminates the need for calibration.

Another important concept in understanding mechanisms of organic reactions is deriving linear free energy relationships (LFERs). In this approach, a series of reactions are conducted, where the dependency of the rate of reactions on electronic and/or steric characteristics of the reagents is characterized, often in varying physical conditions, providing insights into the nature of the transition states involved in the reaction, and so expanding the knowledge about the reaction mechanism. They can also be used to predict the reactivity of new reactants, which can be valuable in designing new chemical reactions or optimizing existing ones. A prime example of a LFER is Hammett analysis, although many other relationships were developed, whilst most recently the field is observing new developments thanks to multivariate regression analysis.[^30^, ^31^, ^32^, ^33^, ^34^, ^35^]

In Hammett analysis, the structure of one of the molecules participating in the reaction (it can be either a reagent or a catalyst) that contains a benzene ring is modified with meta and para positioned substituents. The reaction rates (or equilibrium constants) for so defined derivatives are measured using standard methodology (i.e. initial rates measurements). Then, the reaction rates are compared against tabularized σ Hammett parameters, to determine the effect of substrate substituents on the reaction. For the results to be meaningful, one needs to conduct numerous reactions and analyses thereof, which again, whilst beneficial for the general understanding of the reaction, is often omitted by chemists due to the time‐consuming nature.

To showcase the viability of conducting such analyses in an automated manner using our platform, we focused on pyridine‐catalysed sulphonamide formation between tosyl chloride and a series of eight aniline derivatives. The reactions were monitored with a benchtop Magritek Spinsolve 80 NMR spectrometer using initial rates method, following the reactions over the course of 15 minutes with five different initial concentrations of the aniline derivative. It combined to a total of 40 different experiments and 600 datapoints acquired, with no human intervention required. The reactions were run one arylamine at a time, to prevent deterioration of the pyridine stock solution in dichloromethane, as it is known to form methylenebispyridinium dichloride over time.^[36]^

**Figure 3:**
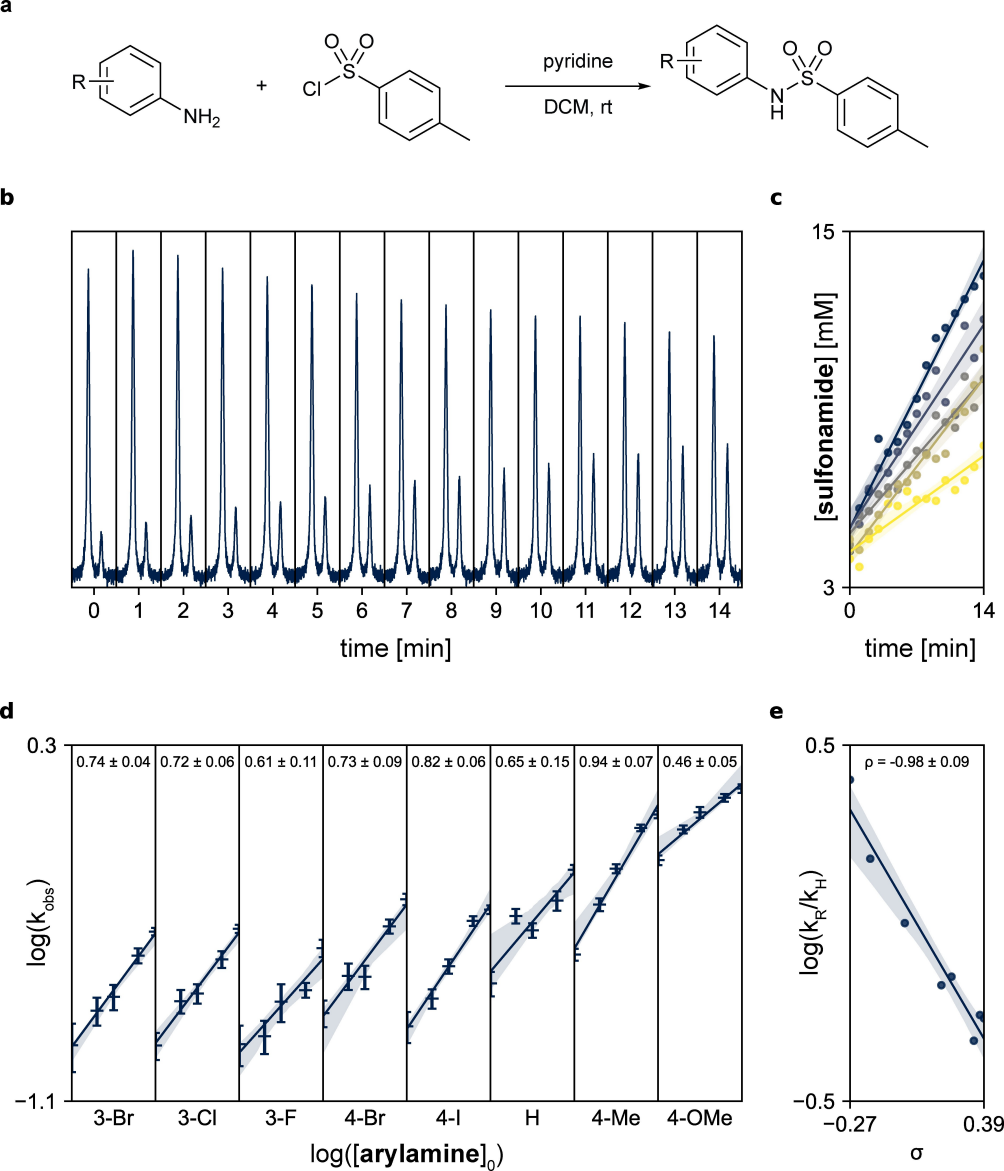
Hammett analysis conducted on pyridine‐catalysed tosylamide formation. [a] Reaction scheme; [b] Waterfall plot for the NMR data acquired using 100 mM aniline as a substrate (the plot has been centred on the diagnostic tosyl methyl region); [c] Integrated data for 5 different initial concentrations of aniline: 100, 75, 50, 37.5 and 25 mM; [d] log‐log plot obtained for aniline experiments; [e] Hammett plot showing a linear correlation between the Hammett parameters and observed reaction rates for differently substituted aniline derivatives.

Exemplar data obtained for non‐substituted aniline is shown in Figure 3b–c, data for the other substrates can be found in the Supporting Information (Section 4.2). Conversion of tosyl chloride to tosylamide was observed as the ratio of methyl signals for tosylamide and all tosyl derivatives combined. Then, an initial rate for each of the experiments was defined as the linear fit of the 15 datapoints. Slopes of these linear fits (representing observed initial reaction rate) were plotted in form of a log‐log plot to determine the order of reaction in the analysed reagent (aniline derivatives). The obtained orders in arylamine vary slightly between different substrates, with the values ranging from 0.46 to 0.94 for the extreme cases (*p*‐anisidine and *p*‐toluidine, respectively). On average the reaction displays an order of approximately 0.7 in arylamine.

One could expect it to be closer to 1.0, as the reaction should intuitively follow an S_N_2 mechanism, however this deviation might be due to the reaction being part of a bigger reaction network, hence making the mechanism more complicated. In additional experiments performed manually, it was observed that reactions in presence of non‐nucleophilic bases such as 2,6‐lutidine or 1,8‐bis(dimethylamino)naphthalene (Proton Sponge) were sluggish, hinting at the importance of pyridine in the mechanism. As presented above, the rapid ability to record kinetic data can accelerate the identification of non‐trivial mechanisms, which can be further studied by expert chemists with additional experiments.

Lastly, observed initial reaction rates for differently substituted anilines were compared with the one for non‐substituted aniline, and the data was plotted as a function of Hammett parameters σ, with the slope of this plot, ρ=−0.98±0.09, reflecting the impact of substituents on this particular reaction. A linear trend throughout the substrate scope and negative value of ρ is in line with expectations.

Having shown that the Chemputer can be used for acquisition of reliable kinetic data in a standardized manner, we next decided to incorporate the reaction monitoring as part of a standard synthetic protocol. It is a common approach when it comes to manual execution of synthetic procedures, routinely in form of thin layer chromatography (TLC). However, automated synthetic protocols usually rely on precise instructions with fixed quantities and reaction times. As it has been shown before, PAT feedback can substantially decrease procedure time for certain classes of reactions, i.e. ones with varying initiation times, such as Grignard reaction.^[20]^ Furthermore, as use of automated platforms such as the Chemputer becomes more widespread, they will be routinely used for execution of new, previously unreported reactions. Gathering kinetic data in an effortless manner for any new reaction run will hopefully hint at unexpected mechanistic traits, expanding our understanding of chemistry, allowing for better design of experiments for the discovery of new reactions.

**Figure 4:**
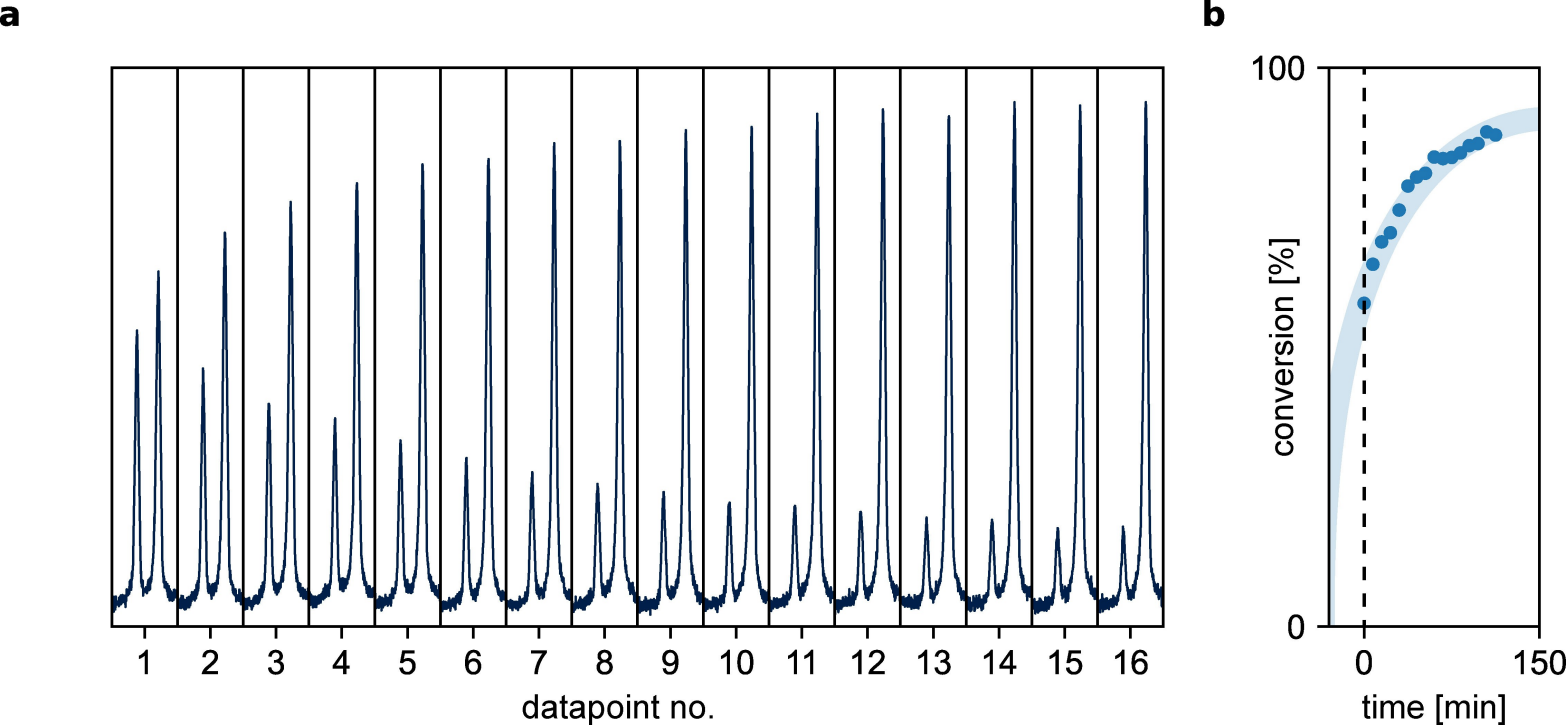
Determining the endpoint of a pyridine‐catalysed tosylation of 4‐bromoaniline monitored by NMR. [a] Horizontally stacked plot for the NMR data throughout the reaction (the plot has been centred on the diagnostic tosyl methyl region); [b] Processed data, showing the conversion of tosyl chloride over time (the blue semi‐transparent line is included for eye guidance). The x‐axis represents time since first measurement, which happened approximately 15 minutes after mixing all the reagents. The measurements were stopped after algorithm has decided that the reaction has reached plateau.

For that purpose, tosylation of 4‐bromoaniline (Figure 3) was conducted on a synthetic scale using an expanded Chemputer setup (See Supporting Information, Section 3.4) and monitored by NMR spectroscopy to determine the endpoint of the reaction. The conversion of tosyl chloride to tosylamide was monitored over time until a plateau was reached, indicating that the reaction had reached completion (Figure 4). To automate the detection of the plateau, a dynamic XDL step was used, which continuously tested whether the difference between a specified number of the most recently acquired spectra has fallen below a certain threshold, analogously to a while loop in programming (See Supporting Information, Section 2.3). This approach allows for precise detection of the reaction endpoint, which is crucial for maximizing yield and purity of the desired product. Once the reaction was nearly complete, which was observed after 16 measurements (approximately two hours into the reaction), a standard work‐up procedure was performed, consisting of extraction, wash, drying and concentrating the sample using a rotary evaporator. The process altogether allowed us to obtain a pure product in an autonomously operating machine, where the time of the reaction was not specified by the user. The plateau determination required *a priori* knowledge of the diagnostic regions for substrate and product, but that can be easily obtained over course of minutes with a quick, small‐scale experiment executed by the chemist. Alternatively, one can envision making the approach even more independent of a human operator by introducing peak‐searching algorithms, which is an area of future study. Additionally, kinetic measurements combined with reaction monitoring permit the development and execution of validated synthetic procedures without the need of PAT. Traditional optimization of chemical reactions focuses on a single outcome of the reaction (i.e., yield, enantiomeric excess). Irrespective of method (“intuition‐based”, one factor at a time (OFAT), Design of Experiments (DoE), or more recently, Bayesian optimization),^[37]^ these approaches require many experiments and rarely provide chemical understanding of the investigated process. Alternatively, acquisition of kinetic data in carefully designed experiments leads to extraction of kinetic parameters (e.g., reaction constants and reagent orders) that establish a chemically descriptive kinetic model. This systematic approach resolves non‐intuitive optimization problems in complicated reactions, i.e., catalyst decomposition, product and/or substrate inhibition.^[4]^

**Figure 5:**
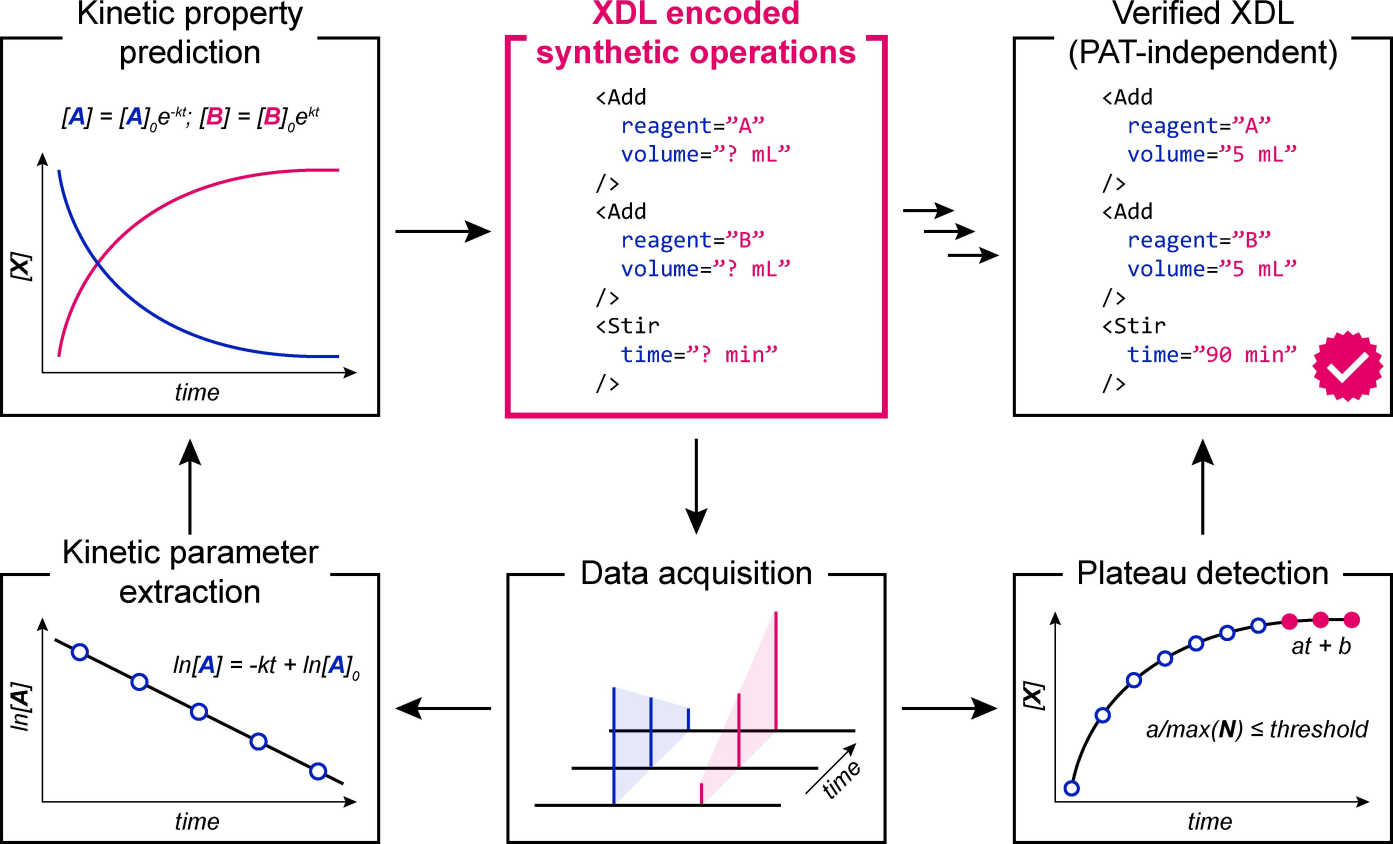
Kinetic modelling combined with reaction monitoring and data analysis on‐the‐fly might allow for dynamic execution of new untested synthetic procedures, but also generation of verified static XDL protocols which can be executed in the future without the need for use of PAT.

As such, we propose a general operational workflow (Figure 5), in which results of initial chemical experiments (encoded in XDL) are used for developing a kinetic model. Further experiments (at different concentrations, reagent ratios, etc.) can be used to improve upon this model in an iterative, automated process. Finally, the improved XDL protocol will undergo empirical validation, whereby a reaction is conducted, analysed, and terminated upon reaching plateau. By determining these reaction parameters in the feedback‐controlled process, a static, verified XDL protocol can be established, which can be reused on different synthetic platforms not bearing PAT, creating a bridge between a dynamic execution of a procedure and a static protocol that can be employed for future use. Overall, this approach can lead to improved efficiency and reproducibility in synthetic chemistry research. This concept is currently under investigation in our laboratory.

## Conclusion

In conclusion, we have demonstrated the ability to encode kinetic measurements in XDL scripts and execute them using Chemputer, as well as the connection between dynamic procedures based on reaction monitoring feedback and validated static protocols. The implementation was possible thanks to the modularity of Chemputer, which allows for straightforward expansion of the setup with different process analytical technologies (PAT). To showcase this functionality, we have used on‐line UV/Vis and NMR spectroscopy and followed kinetic experiments using initial rate methods and variable time normalization analysis (VTNA). Furthermore, we have shown how our approach can be applied to analyses not only of single reactions, but classes of reactions, as exemplified with the Hammett analysis. Finally, we discussed the benefits of executing synthetic procedures with feedback from PAT, and how these dynamic procedures lead to static synthetic protocols, that can be reused in the future without the need to use external analytics. We envision that our approach will allow chemists to acquire kinetic data more routinely, with the generated data forming a database of use for the Machine Learning community, eventually allowing us to better understand reactivity patterns. Ultimately, we envision that adoption of standardized methods such as presented herein will make kinetic data acquisition more common and reliable, which can have only beneficial impact for all fields of chemistry.

## Supporting Information

The Supporting Information contains information about all the experiments performed and the relevant data produced, description of the analytical XDL steps and the plateau detection algorithm. Supporting Information dataset is archived on Zenodo. 10.5281/zenodo.10368978.

## Conflict of interest

The authors declare no conflict of interest.

1

## Supporting information

As a service to our authors and readers, this journal provides supporting information supplied by the authors. Such materials are peer reviewed and may be re‐organized for online delivery, but are not copy‐edited or typeset. Technical support issues arising from supporting information (other than missing files) should be addressed to the authors.

Supporting Information

## Data Availability

The data that support the findings of this study are available in the supplementary material of this article.
