# Supplementary material for: Reaction Kinetics using a Chemputable Framework for Data Collection and Analysis
Source: Angew Chem Int Ed Engl. 2024 Jan 24;63(9):e202315207. doi: 10.1002/anie.202315207 (PMC11497221; doi:10.1002/anie.202315207)
Supplement: Supplementary file 1 — Supporting Information [file ANIE-63-e202315207-s001.pdf]

Angewandte Chemie
Eine Zeitschrift der Gesellschaft Deutscher Chemiker

Supporting Information

Reaction Kinetics using a Chemputable Framework for Data Collection and Analysis

*B. M. Matysiak, D. Thomas, L. Cronin**

SUPPORTING INFORMATION

Reaction Kinetics using a Chemputable Framework for Data Collection and Analysis

Bartosz M. Matysiak, Dean Thomas, Leroy Cronin*

School of Chemistry, University of Glasgow, Glasgow, G12 8QQ,

Contents

1. Materials and Instrumentation 3
  1.1. Materials 3
  1.2. Chemputer Platform 3
  1.3. Offline NMR Measurements 3
  1.4. General Procedure For Online UV-Vis Measurements 3
  1.5. General Procedure For Online NMR Measurements 4
2. Custom XDL Steps 4
  2.1. XDL Step For NMR Data Acquisition 4
  2.2. XDL Step For UV-Vis Data Acquisition 4
  2.3. NMR Reaction Monitoring 5
3. Experimental Procedures 9
  3.1. Inverse Electron-Demand Diels-Alder (UV-Vis) 9
  3.2. Metal Complexation (UV-Vis) 10
  3.3. Arylamine Tosylation – Hammett Analysis (NMR) 11
  3.4. 4-Bromoaniline Tosylation – Reaction Monitoring (NMR) 12
4. Analytical Data 14
  4.1. Analytical Workflow For Variable Time Normalization Analysis 14
  4.2. Arylamine Tosylation – Hammett Analysis (NMR) 17
  4.3. Arylamine Tosylation – Reaction Monitoring (NMR) 25
5. References 26

1. Materials and Instrumentation

1.1. Materials

The reagent-grade chemicals were obtained from Fluorochem, Sigma-Aldrich and TCI. The aniline derivatives were purified by recrystallization or distillation and stored in a desiccator prior to use. Other reagents were used as obtained without further purification. Solvents were purchased from several departmental suppliers, Honeywell, Fisher and Sigma-Aldrich.

1.2. Chemputer Platform

The pumps, valves and frames are standardised pieces of equipment designed by the Cronin Group and assembled as required. Fittings, adaptors, tubing and other commercially standardised parts are purchased from suppliers including RS Components. The exact part numbers for a standardised platform alongside extensive build instructions are readily available.[1]

1.3. Offline NMR Measurements

NMR measurements were performed with Bruker Avance III HD 600 spectrometer operating at 600.1 and 150.9 MHz for ^{1}H and ^{13}C, respectively. Spectra were collected at 298 K, chemical shifts are reported in ppm and were calibrated for the (residual) NMR solvent signal (multiplicities are given as s: singlet, d: doublet, t: triplet, q: quartet, m: multiplet, with coupling constants reported in Hz). The spectra were processed with MestReNova 14.0.0.

1.4. General Procedure For Online UV-Vis Measurements

UV-Vis spectra were acquired with a DH-2000 light source and a FIA-Z-SMA 905 PEEK (10 mm path length) flowcell from Ocean Optics, connected by fibre optics to an Avantes AvaSpec-DUAL 4096 spectrophotometer.

The light source was manually switched on prior to execution of experiments employing UV-Vis spectrophotometry and manually turned off afterwards. The reference spectra for required solvents were acquired manually just before starting experiments on given day and used for further processing of the raw data.

All the experiments were done in triplicate. The reactions were run at concentrations such that the detector of UV-Vis apparatus did not get oversaturated, albeit our setup does allow for diluting the sample prior to the measurement.

1.5. General Procedure For Online NMR Measurements

NMR measurements were performed with a benchtop Magritek Spinsolve 80 spectrometer operating at 80 MHz for ^{1}H. Spectra were collected at 298 K, chemical shifts are reported in ppm and were calibrated for the solvent signal of non-deuterated solvent - dichloromethane (assuming shift of 5.30 ppm for approximation). The spectra were processed with MestReNova 14.0.0.

The benchtop Magritek Spinsolve 80 spectrometer was equipped with a flowcell, the bottom of which was connected to the Chemputer backbone. During kinetic measurements, the top of the flowcell was connected directly to waste. During reaction monitoring experiments, the top of the flowcell was connected to the Chemputer backbone, allowing transfer of material back to the reactor. Shortly prior to using the machine, a QuickShim protocol was executed to ensure a satisfactory signal to noise ratio.

2. Custom XDL Steps

The platform (a standard implementation of Chemputer as described by Rohrbach et al.[1]) was operated using XDL 2.0 (as described at https://croningroup.gitlab.io/chemputer/xdl/ as of August 2023). The analytical extensions in form of benchtop NMR and UV-Vis spectrometers were connected as additional modules, requiring additional XDL steps to operate them.

2.1. XDL Step For NMR Data Acquisition

The Magritek Spinsolve spectrometer was operated using a **RunProton** step. The step is based on the Magritek Spinsolve API and allows for specifying the type of ^{1}H NMR experiment (i.e., controlling the number of scans).

2.2. XDL Step For UV-Vis Data Acquisition

The Avantes spectrophotometer was operated using a **RunUV** XDL step, based on the Python API available on the Avantes website (https://avantesusa.com/ as of August 2023). The step leads to acquisition of spectrum and dumping it under a specified path. As no decision-making based on UV-Vis readout was done, data processing was not included in that or other steps.

2.3. NMR Reaction Monitoring

For determining the endpoint of a reaction, two new XDL steps were developed: a ***ProcessNMR*** step and a ***MonitorPlateau*** step.

2.3.1. <ProcessNMR/>

ProcessNMR is a XDL step processing the last acquired ^{1}H NMR spectrum. As arguments it takes chemical shifts (in form of tuples with upper and lower limit) of the signal of interest and a reference signal, theoretical ratio between them, chemical shift of the solvent-signal, and ID of a XDL Parameter to store the outcome of data processing in. The reference signal might be an added qNMR standard, or a signal coming from one of the reagents involved in the reaction. The theoretical ratio is a parameter that applies only in case of qNMR, which was not used throughout this work, and so is not important for the rest of this document. The solvent signal is the signal coming from solvent of the reaction and is determined as the highest signal in the spectrum. The XDL Parameters are a functionality of XDL allowing for storing and reading numerical values in a XDL procedure. ***ProcessNMR*** loads the last acquired NMR spectrum, applies standard processing procedure to it, integrates the signal and reference areas, and stores the ratio between them in a specified XDL Parameter. Typically, it will be used right after acquiring a spectrum, e.g., by ***RunProton.***

2.3.2. <MonitorPlateau/>

MonitorPlateau is a XDL step that allows for determining the endpoint of a reaction or other processes (for instance titration, dissolution, breaking emulsion, etc.). It is technique-agnostic i.e., it tracks changes in the numerical values obtained from processing the raw measurement data, and so can deal with data coming from any type of measuring device (like NMR spectrometer, but also conductivity sensor, IR probe, thermometer, camera, etc.). As arguments it takes: XDL Parameter ID, name of file in which it ought to store the data, number of datapoints and threshold value. The latter 2 arguments are parameters to be used by the algorithm that decides whether the endpoint has been reached (i.e., the data started plateauing). Additionally, it requires maximum number of datapoints – which allows for terminating the step after a certain number of datapoints if the plateau has not been detected up to this point.

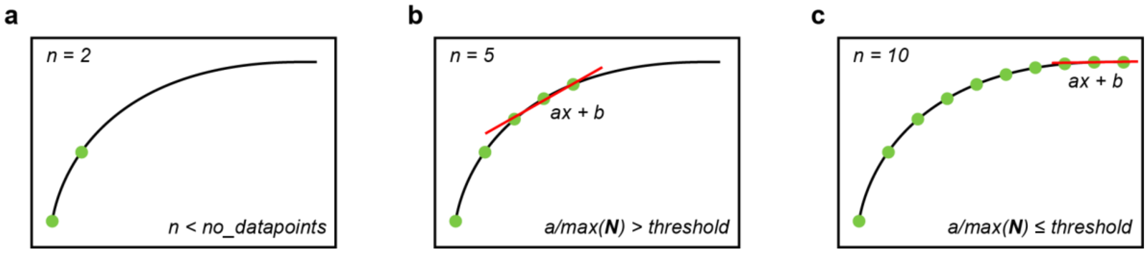

Figure S1 - The plateau detection algorithm used in conjunction with NMR data. [a] When the number of datapoints is below a set minimal **no_datapoints**, the algorithm decides that the reaction still proceeds; when number of datapoints is equal to or above **no_datapoints**, the algorithm tests the slope of linear regression for a specified number (**no_datapoints**) of last datapoints divided by the maximum value of the whole dataset (max(N)) against a user-specified threshold. If the value obtained is above the threshold (b), the algorithm decides that the reaction still proceeds; if the value falls below the threshold (c), the algorithm determines it to be the endpoint of the reaction and exits the <Repeat/> loop.

Detecting the plateau is achieved by means of the following algorithm: firstly, the number of datapoints (which are stored in the data file) is compared against the **no_datapoints** argument. If the number of datapoints is smaller than **no_datapoints**, the algorithm sends the step into the next loop. If the number of datapoints exceeds **no_datapoints**, the slope of linear regression applied to the last **no_datapoints** datapoints, normalized by dividing it by the maximum value of the whole dataset is compared against the **threshold**. If it is above the threshold, the plateau has not yet been detected and the algorithm sends the step into the next loop. If the change falls below **threshold**, the algorithm decides that the plateau has been reached, and the XDL step returns a XDL constant **DONE**, which causes termination of the **Repeat** loop. On top of that, if the number of acquired datapoints exceeds **max_no_datapoints** without detecting the plateau, the **Repeat** loop is also terminated. The whole workflow can be represented in terms of pseudocode in **Figure S2**:

```
N = whole data acquired until now
n = number of datapoints required for LinearRegression
    (operates on last n datapoints)

DEF RESULT:
    RETURN LinearRegression(n).SLOPE / MAX(N)

WHILE True:
    Move reaction to NMR
    Run NMR analysis
    Process NMR spectrum
    IF number_of_datapoints < n:
        RETURN True
    ELIF RESULT > THRESHOLD:
        RETURN True
    ELIF number_of_datapoints > max_number_of_datapoints:
        RETURN False
    ELSE:
        RETURN False
```

Figure S2 – Pseudocode for the ***MonitorPlateau*** workflow.

The step is to be used as a part of bigger XDL block. It is placed inside of a **Repeat** loop with unspecified number of repeats, together with a XDL step that provides a new numerical value with every repeat.

In the subsequent example (**Figure S3**), it is preceded by an **NMRAnalysis** blueprint (that contains **RunProton** step, not shown) and a **ProcessNMR** step. The two steps combined provide a numerical value representing the conversion of the substrate, which is stored in a XDL Parameter **conversion**. **MonitorPlateau** reads **conversion** and **datafile.txt** (that contains prior datapoints), adds it to a new line in **datafile.txt**, and applies the aforementioned algorithm to the data. Once the **MonitorPlateau** step exits the **Repeat** loop, the rest of the XDL procedure is executed afterwards, *i.e.*, workup of the reaction.

```xml
<Repeat>
    <NMRAnalysis
        experiment_name="N-tosyl-4-bromoaniline"
        sample="reactor"
        nmr_forward="nmr_forward"
        nmr_backward="nmr_backward"
        pump_bottom="Pump_D"
        pump_top="Pump_C"
        flush_gas="flask_air"
    />
    <ProcessNMR
        signal="(2.40, 2.20)"
        reference="(2.65, 2.40)"
        theoretical_ratio="1"
        parameter_id="par.conversion"
        parameter_type="float"
        solvent_signal="5.30"
    />
    <MonitorPlateau
        data_file="datafile.txt"
        no_datapoints="8"
        max_no_datapoints="70"
        threshold="0.001"
        parameter_id="par.conversion"
    />
</Repeat>
```

Figure S3 – XDL snippet representing use of **MonitorPlateau** in conjunction with NMR acquisition (within **NMRAnalysis** blueprint) and processing *via* **ProcessNMR**.

3. Experimental Procedures

3.1. Inverse Electron-Demand Diels-Alder (UV-Vis)

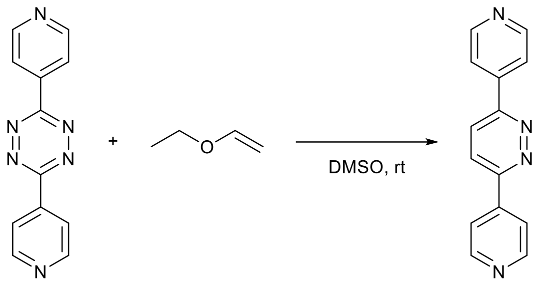

Stock solutions of 3,6-di(4-pyridyl)-1,2,4,5-tetrazine (from now on called “tetrazine”) and ethyl vinyl ether (EtOVin) in DMSO were prepared shortly prior to use at 5 and 500 mM, respectively. For each of the experiments, adequate volumes of the stock solutions and solvent were transferred to a reactor (a 14 mL capped vial with septum) to give total of 10 mL of reaction mixture with initial concentrations of the reagents being: 1.5 mM tetrazine and 100/150/200 mM EtOVin. After setting up the reaction, the reaction mixture was transferred to the flowcell and back to the reactor to prime the flowcell. Afterwards, the reaction mixture was transferred to the flowcell, the UV-Vis spectrum was measured, and the reaction mixture was sent back to the reactor 20 times with a waiting period after the liquid transfer and spectrum acquisition of 30 seconds. After acquisition of the data, the reactor and flowcell would be emptied and washed twice with DMSO, followed by setting up the next experiment. The absorbance at 540 nm was used for processing the data.

3.2. Metal Complexation (UV-Vis)

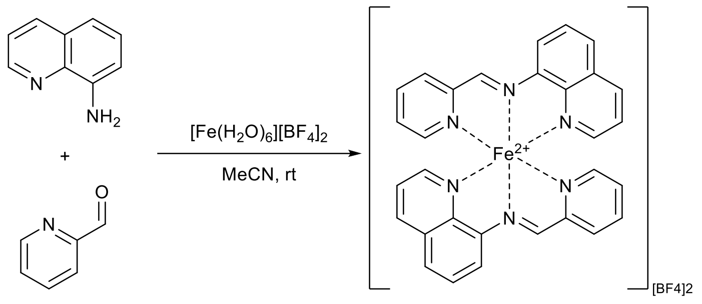

Stock solutions of $Fe(BF_4)_2$ hexahydrate, 8-aminoquinoline and 2-formylpyridine in acetonitrile (MeCN) were prepared shortly prior to use at 0.5, 1.0 and 1.0 mM, respectively.

For the initial experiment, adequate volumes of the stock solutions and solvent were transferred to a reactor (a 14 mL capped vial with septum) to give total of 9 mL of reaction mixture with initial concentrations of the reagents being: 83.3, 167 and 167 μM, respectively. After setting up the reaction, the reaction mixture was transferred to the flowcell and back to the reactor to prime the flowcell. Afterwards, the reaction mixture was transferred to the flowcell, the UV-Vis spectrum was measured, and the reaction mixture was sent back to the reactor 120 times, with a waiting period after the liquid transfer and spectrum acquisition of 60 seconds. After acquisition of the data, the reactor and flowcell would be emptied and washed twice with MeCN, followed by setting up the next experiment.

In the next experiments, each of the reagents was used in a slight (1.2 eq) excess as compared to the initial experiment, whilst keeping the other reagent concentrations (and total reaction volume) the same. The data acquisition was done in an analogous manner.

The absorbance at 660 nm was used for processing the data.

3.3. Arylamine Tosylation – Hammett Analysis (NMR)

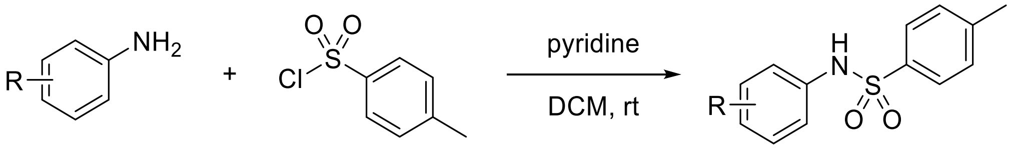

For each of the arylamines, the following procedure was applied:

Stock solutions of all reagents in DCM were prepared shortly prior to use with the following concentrations: 300 mM arylamine, 110 mM tosyl chloride (TsCl), 300 mM pyridine. For each of the experiments, adequate volumes of the stock solutions were transferred to a reactor (a 14 mL capped vial with septum) to give total of 12 mL of reaction mixture with initial concentrations of the reagents being: 100 mM pyridine, 36.7 mM TsCl and 100/75/50/37.5/25 mM arylamine. After setting up the reaction, the reaction mixture was transferred to the flowcell, where it was kept for 15 minutes, allowing for acquisition of 15 NMR spectra with 4 scans per spectrum. After acquisition of the data, the reactor and flowcell would be emptied and washed twice with DCM, followed by setting up the next experiment.

3.4. 4-Bromoaniline Tosylation – Reaction Monitoring (NMR)

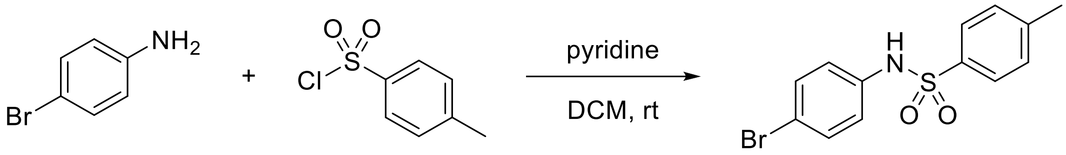

A 50 mL RBF was charged with 500 mg of 4-bromoaniline (1 eq.) and 570 mg of tosyl chloride (1.02 eq.). To that RBF, 25 mL of DCM containing 260 µL of pyridine (1.1 eq.) was added. After that 10 mL of the reaction mixture would be transferred to the flowcell, followed by acquisition of NMR spectrum and sending the reaction mixture back to the RBF. The NMR spectra would be acquired every ~450 seconds. After 16 spectra were acquired, the algorithm has decided that the reaction has reached plateau. After that, the contents of the RBF were sent to a separator and washed with 25 mL of saturated NH_4Cl. The aqueous phase was extracted with 2x20 mL of DCM, and combined organic layers were washed with 25 mL of water and 25 mL of brine. After that, combined organic layers were sent through a cartridge packed with $MgSO_4$ to a rotary evaporator and the solvent removed *in vacuo*. The product was obtained as an off-white/pink solid – 824 mg (87%). The NMR spectrum was in accordance with literature.[1]

^{1}H NMR (600 MHz, CDCl3) δ 7.64 (d, *J* = 8.2 Hz, 2H), 7.35 (d, *J* = 8.5 Hz, 2H), 7.24 (d, *J* = 8.2 Hz, 2H), 6.95 (d, *J* = 8.5 Hz, 2H), 2.39 (s, 3H).

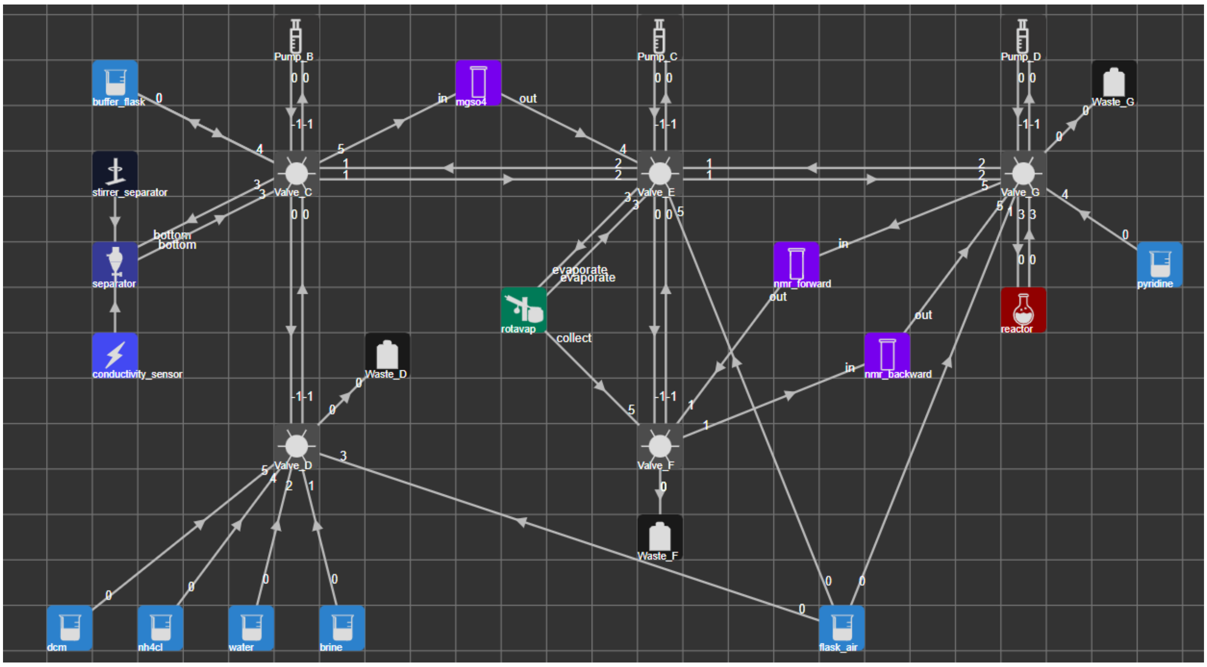

Figure S4 - Graph of the platform used for the reaction monitoring experiment.

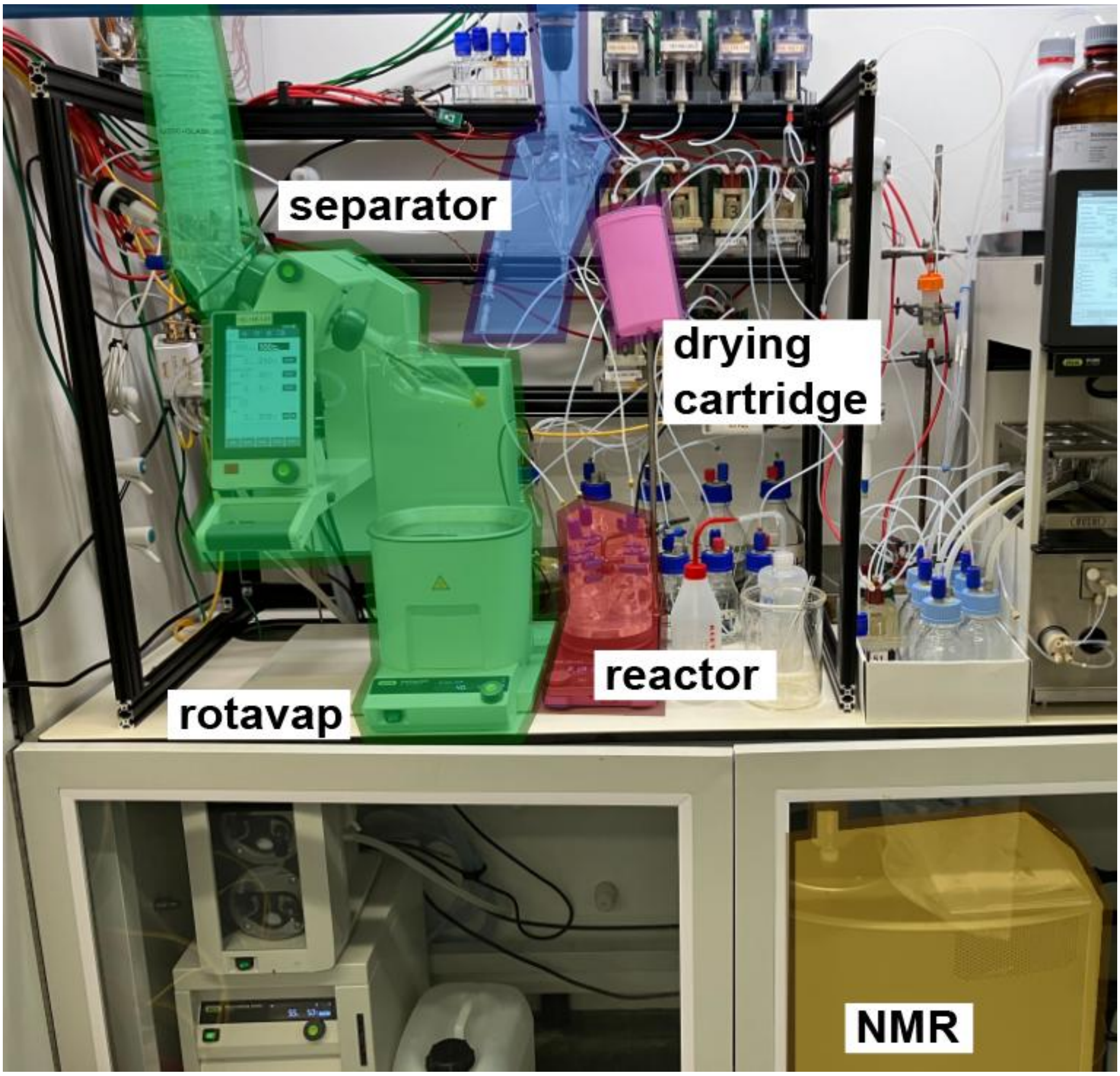

Figure S5 - Photo of the platform used for the reaction monitoring experiment, with crucial elements of the platform highlighted.

4. Analytical Data

4.1. Analytical Workflow For Variable Time Normalization Analysis

The analysis was done with three approximations:

1) Only the reaction product is absorbing at the wavelength used for analysis (660 nm).
2) The substrates at any time are present predominantly in the form of starting materials or the reaction product.
3) The final recorded absorbance represents full conversion of substrates to product in the stoichiometric experiment.

Based on these assumptions, the recorded absorbance at 660 nm is converted to substrate concentration time series. This data is then submitted to Variable Time Normalization Analysis, as described in detail in the original study from Burés.[2]

Briefly, for every next two datapoints, the average substrate concentration raised to the power of order being tested and multiplied by time difference between these two datapoints was calculated. These values were then cumulatively summed and used as the new abscissa axis. The absorbance at 660 nm was plotted against this new abscissa axis series for the stoichiometric and excess experiment for a series of different orders being tested. The order for which the two lines are visually overlapping the most is chosen as the one best describing the order of the reaction in the excess reagent.

The results of such analysis are shown in **Figure S6** and **Figure S7**. The Jupyter notebook used for conducting this analysis can be found in the appendix in folder /experiments/2a.

4.1.1. 2-Formylpyridine VTNA

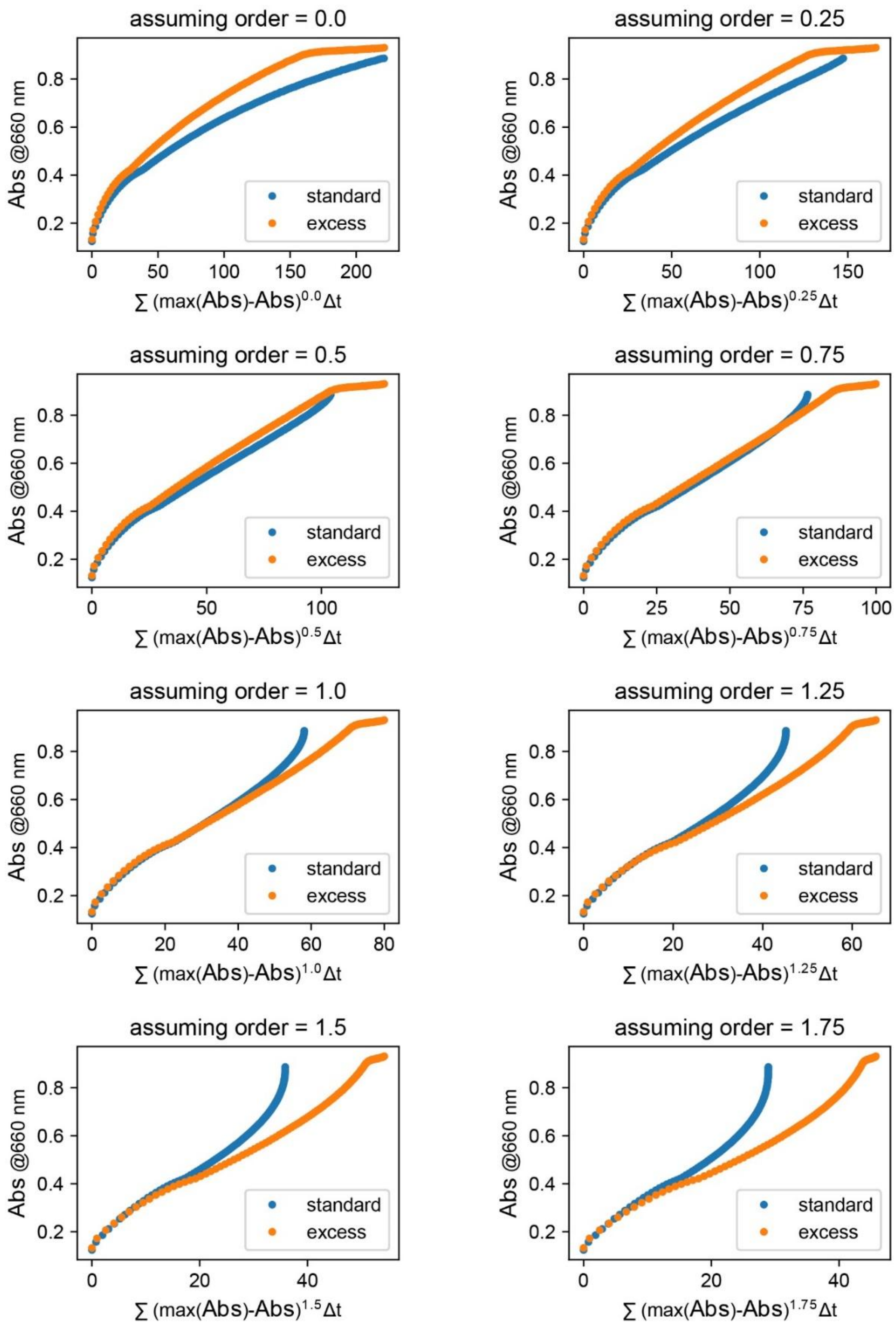

Figure S6 – Plots showing the results of VTNA analysis for 2-formylpyridine as described in Section 4.1. The highest overlap between standard and excess experiment was observed around order = 1.

4.1.2. 8-Aminoquinoline VTNA

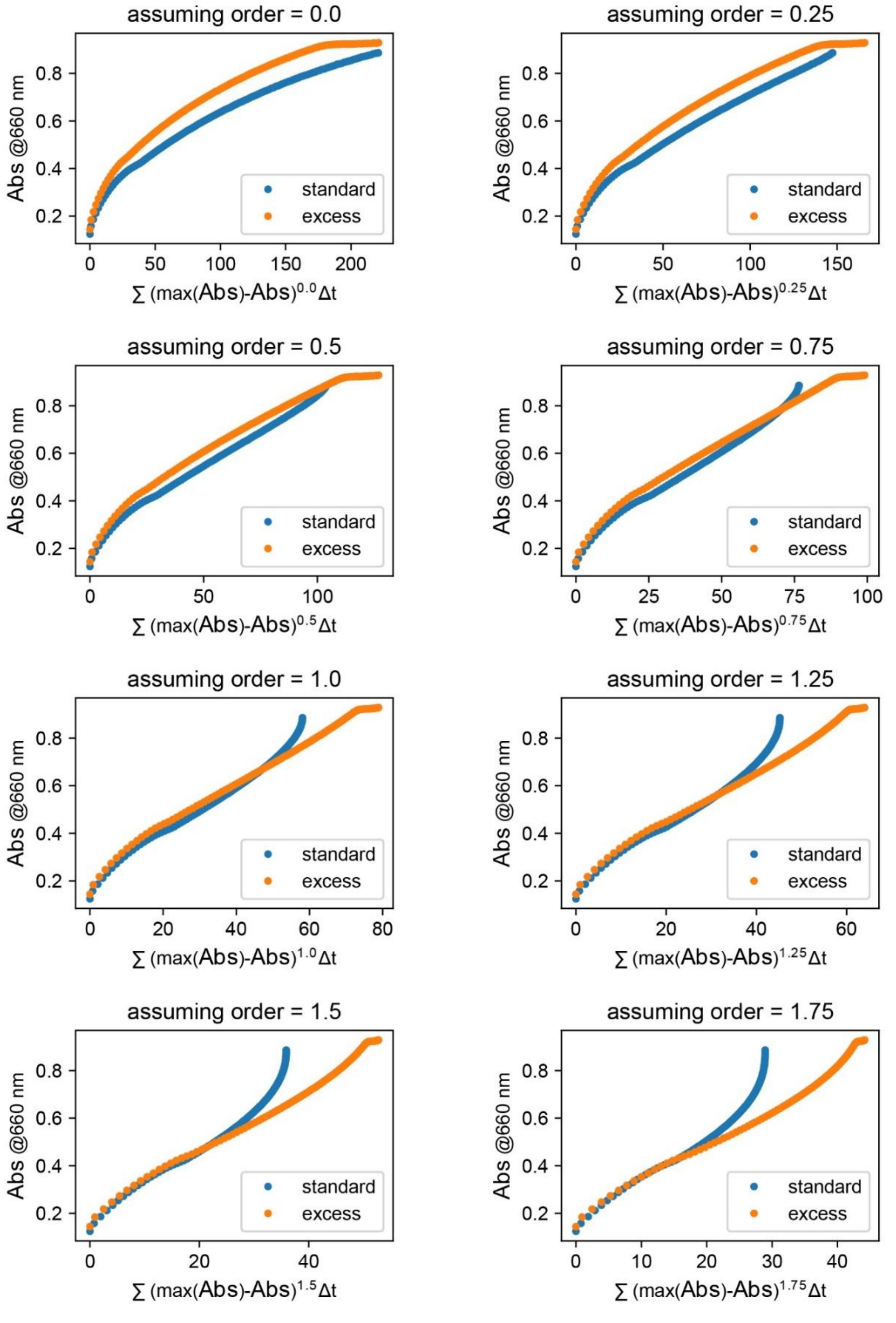

Figure S7 – Plots showing the results of VTNA analysis for 8-aminoquinoline as described in Section 4.1. The highest overlap between standard and excess experiment was observed around order = 1.

4.2. Arylamine Tosylation – Hammett Analysis (NMR)

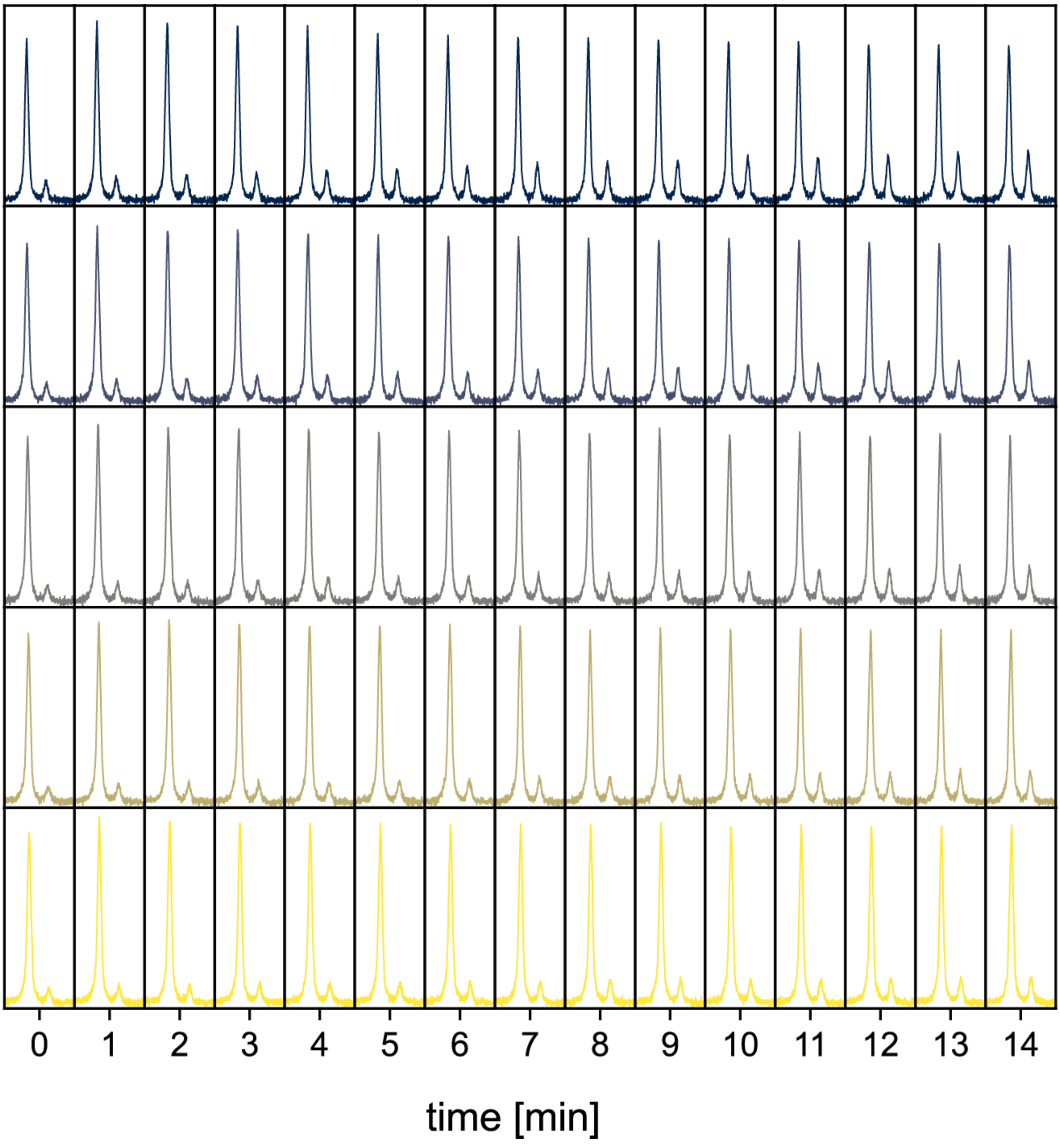

Figure S8 - Inset of diagnostic region in the ^{1}H NMR spectrum of the 75 experiments executed for 3-bromoaniline. Rows correspond to different concentrations: 100 mM, 75 mM, 50 mM, 37.5 mM and 25 mM from top to bottom.

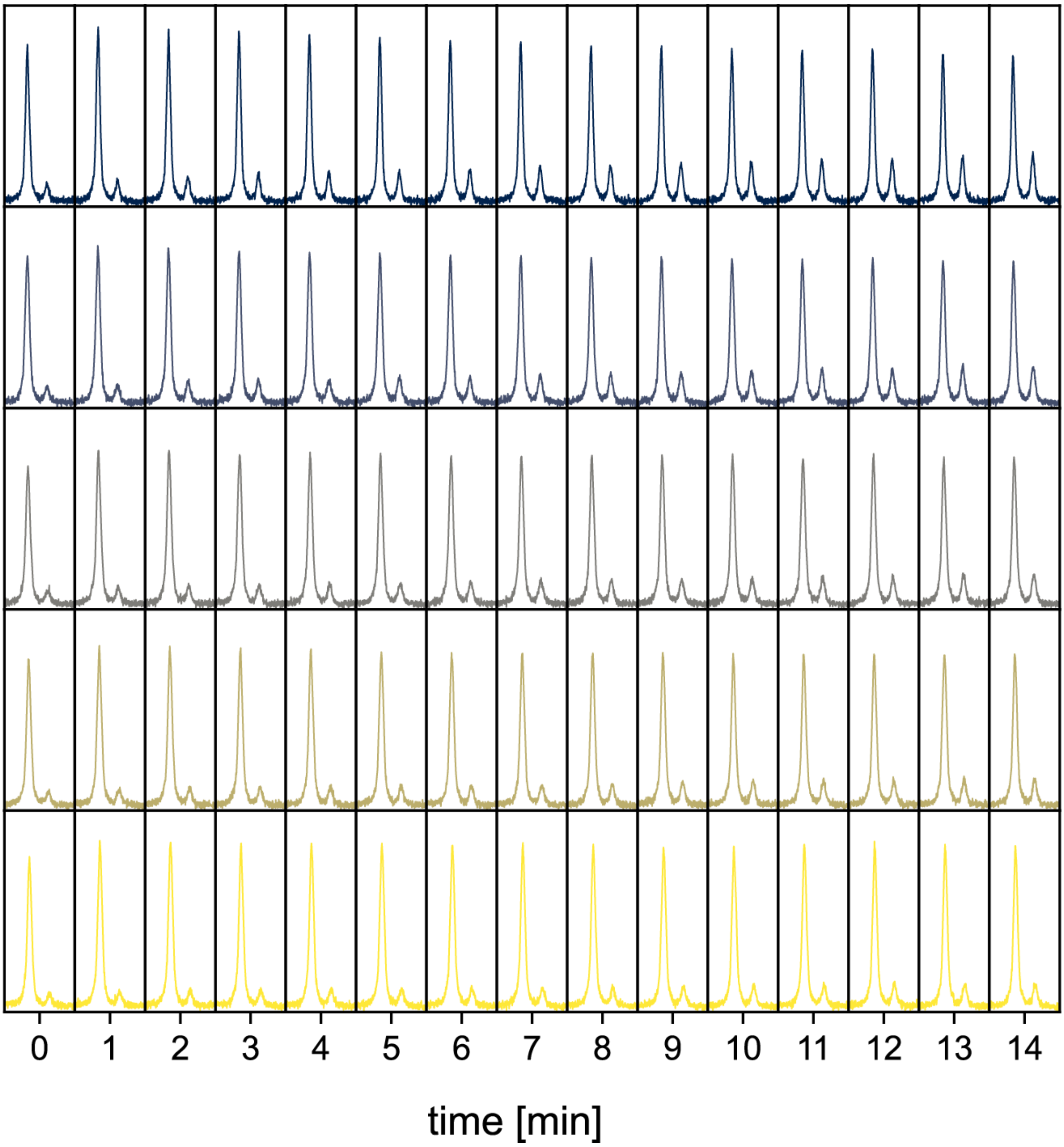

Figure S9 - Inset of diagnostic region in the ^{1}H NMR spectrum of the 75 experiments executed for 3-chloroaniline. Rows correspond to different concentrations: 100 mM, 75 mM, 50 mM, 37.5 mM and 25 mM from top to bottom.

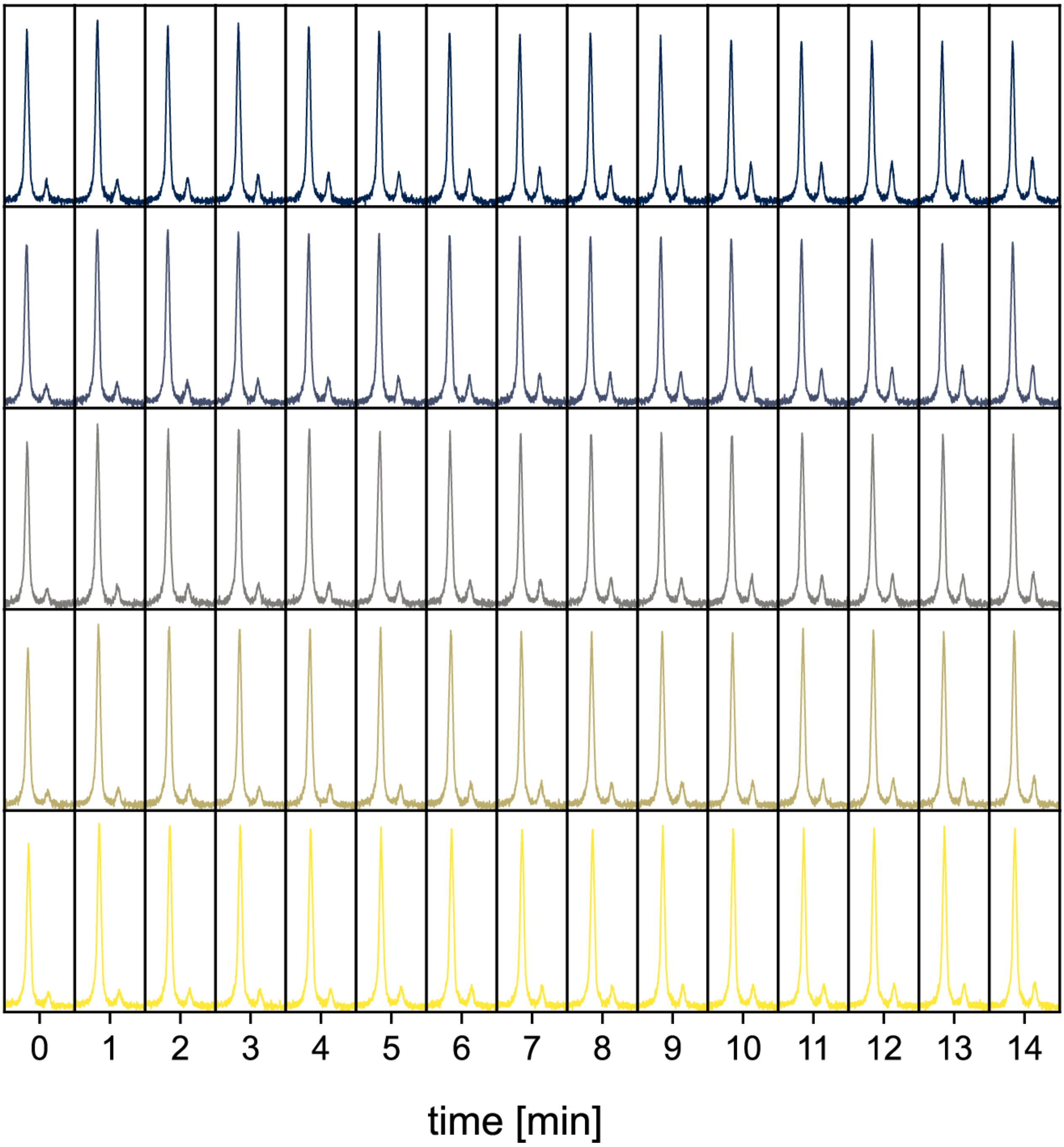

Figure S10 - Inset of diagnostic region in the ^{1}H NMR spectrum of the 75 experiments executed for 3-fluoroaniline. Rows correspond to different concentrations: 100 mM, 75 mM, 50 mM, 37.5 mM and 25 mM from top to bottom.

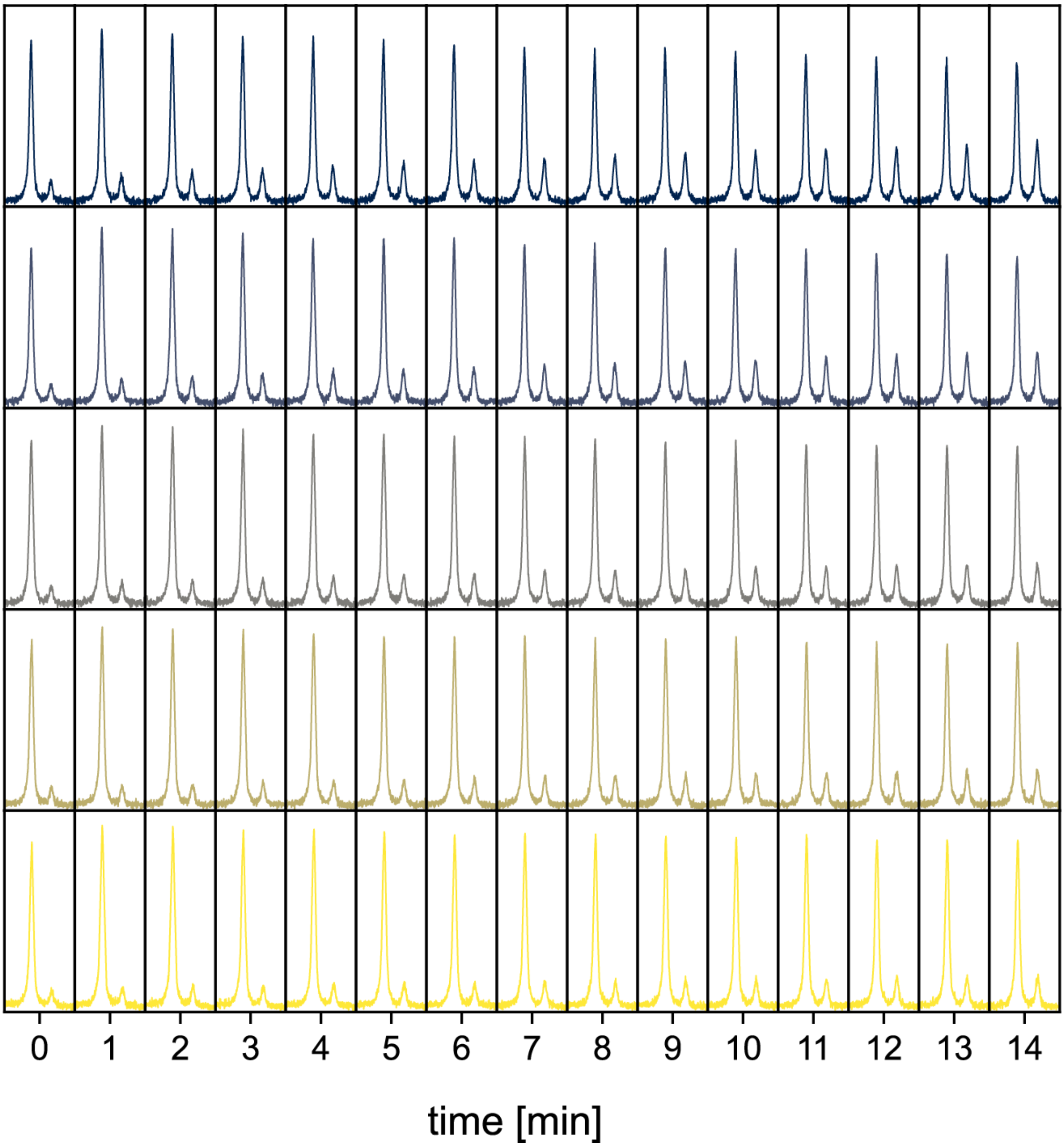

Figure S11 - Inset of diagnostic region in the ^{1}H NMR spectrum of the 75 experiments executed for 4-bromoaniline. Rows correspond to different concentrations: 100 mM, 75 mM, 50 mM, 37.5 mM and 25 mM from top to bottom.

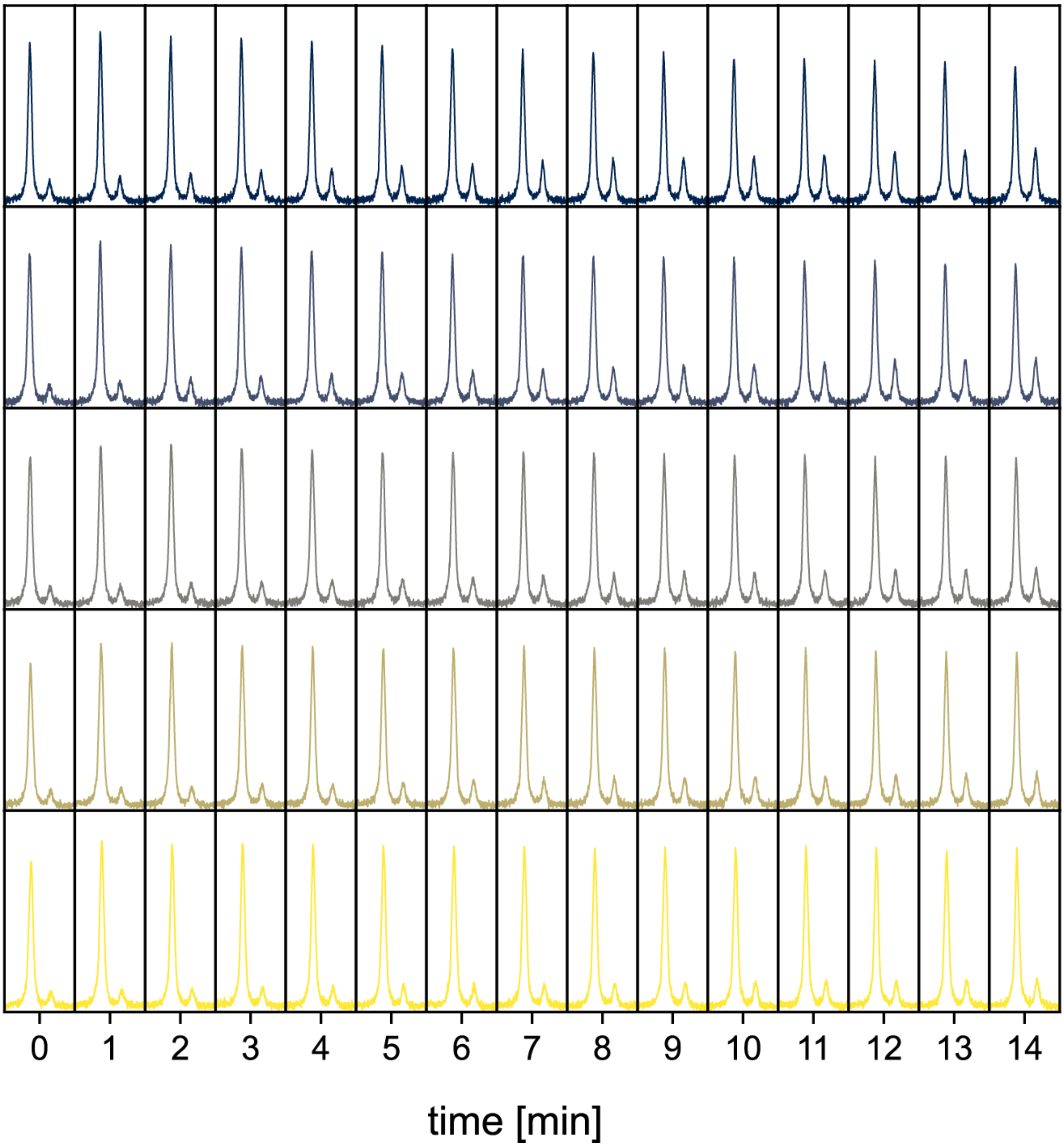

Figure S12 - Inset of diagnostic region in the ^{1}H NMR spectrum of the 75 experiments executed for 4-iodoaniline. Rows correspond to different concentrations: 100 mM, 75 mM, 50 mM, 37.5 mM and 25 mM from top to bottom.

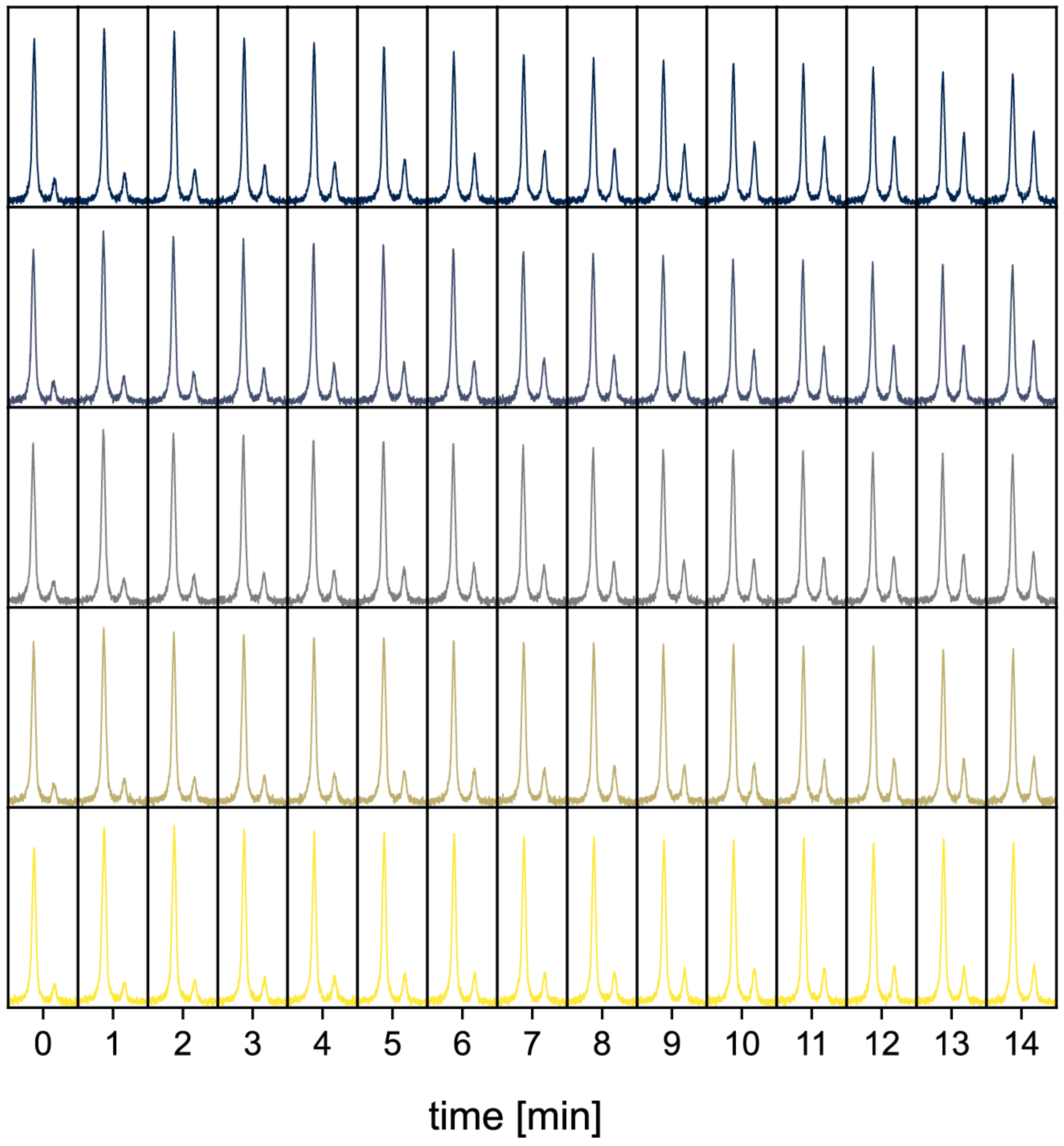

Figure S13 - Inset of diagnostic region in the ^{1}H NMR spectrum of the 75 experiments executed for aniline. Rows correspond to different concentrations: 100 mM, 75 mM, 50 mM, 37.5 mM and 25 mM from top to bottom.

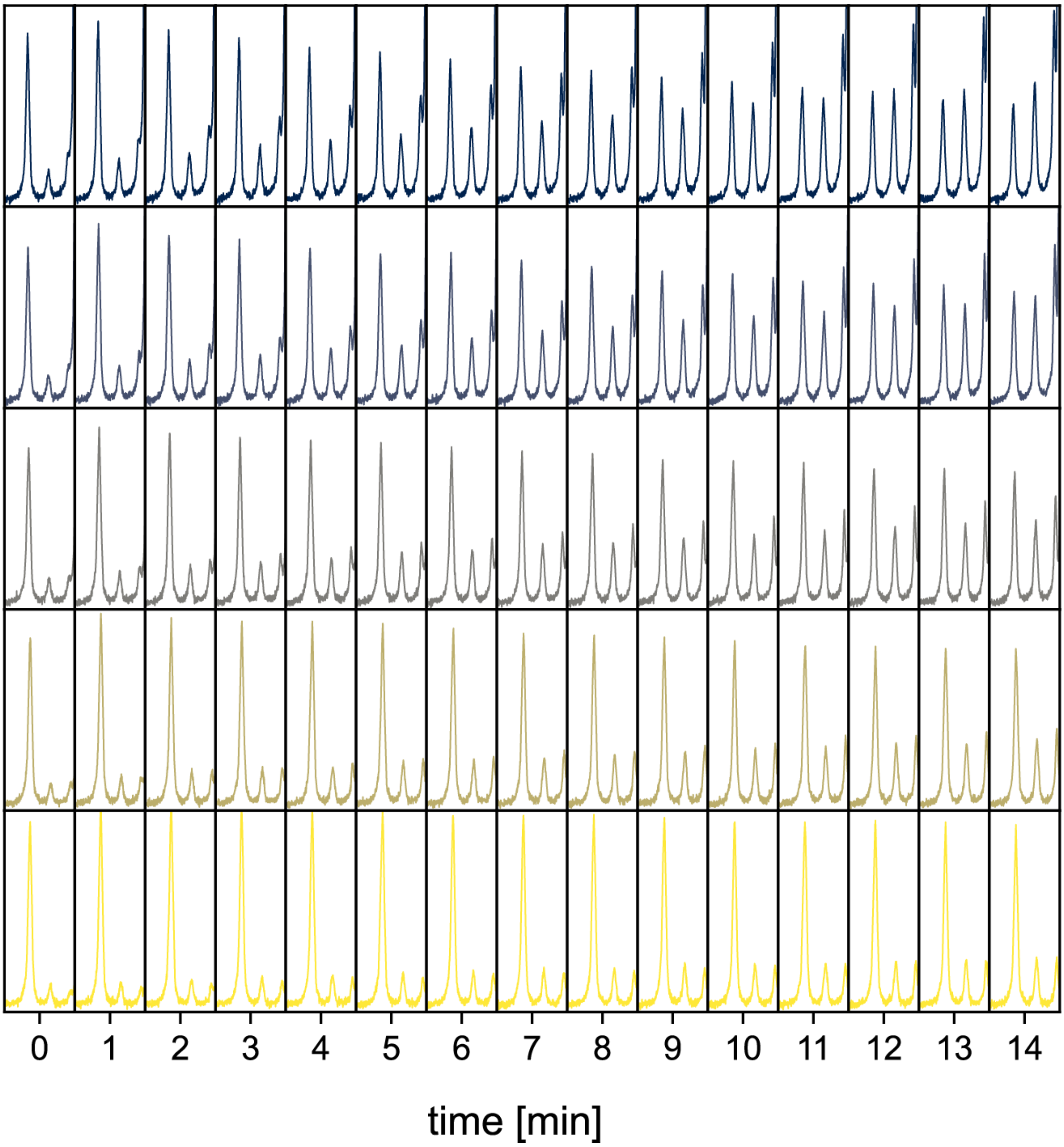

Figure S14 - Inset of diagnostic region in the 1H NMR spectrum of the 75 experiments executed for 4-methylaniline. Rows correspond to different concentrations: 100 mM, 75 mM, 50 mM, 37.5 mM and 25 mM from top to bottom.

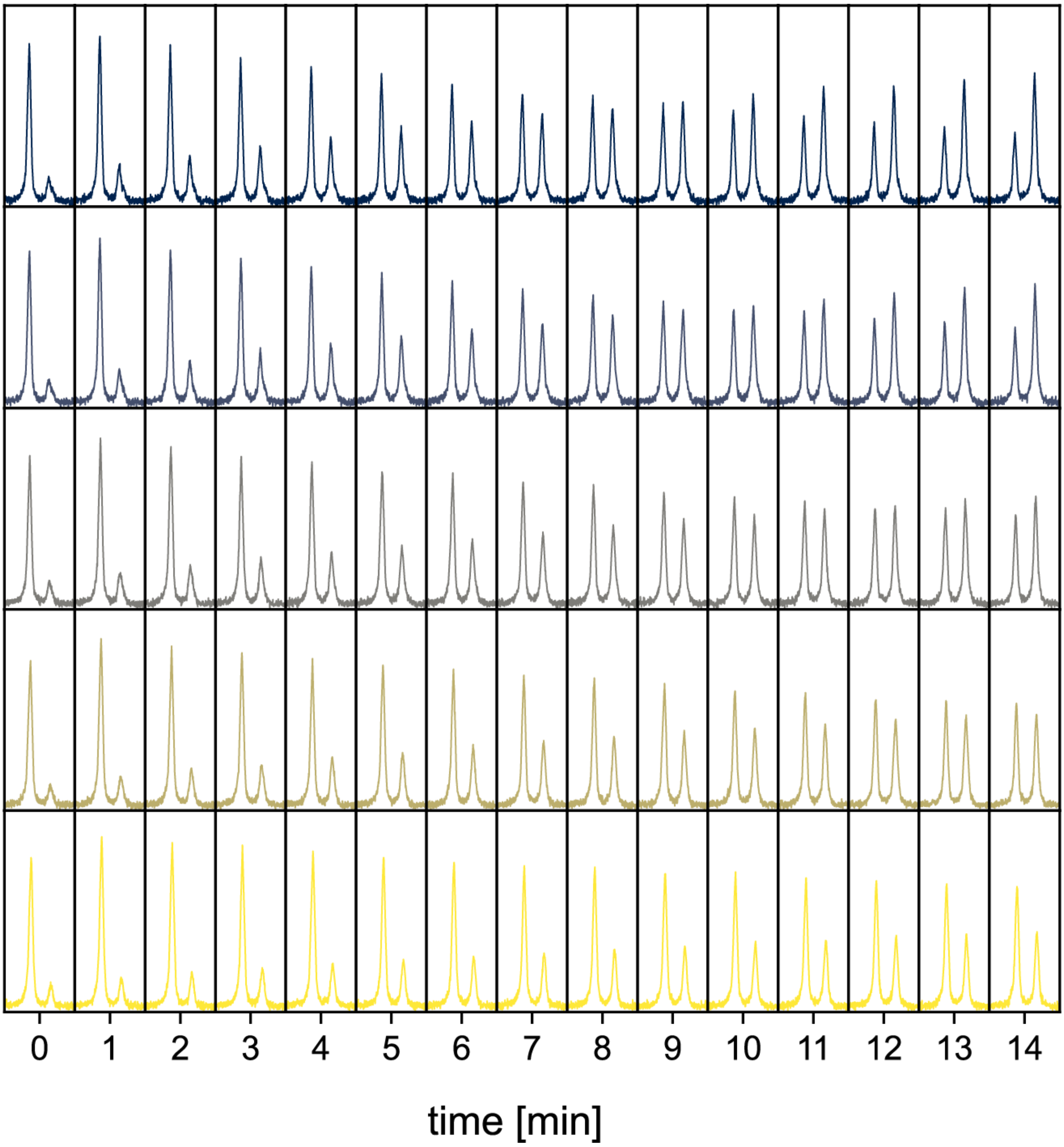

Figure S15 - Inset of diagnostic region in the ^{1}H NMR spectrum of the 75 experiments executed for 4-methoxyaniline. Rows correspond to different concentrations: 100 mM, 75 mM, 50 mM, 37.5 mM and 25 mM from top to bottom.

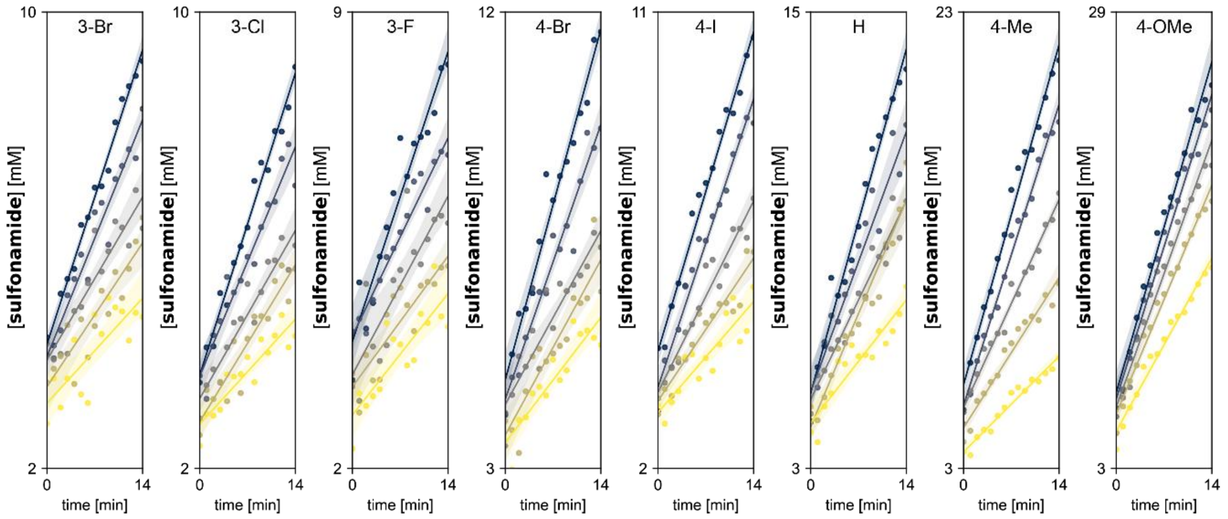

Figure S16 - Combined integrated data for 5 different initial concentrations (100 mM, 75 mM, 50 mM, 37.5 mM and 25 mM) of 8 different arylamines (ordered from left to right based on Hammet σ value).

4.3. Arylamine Tosylation – Reaction Monitoring (NMR)

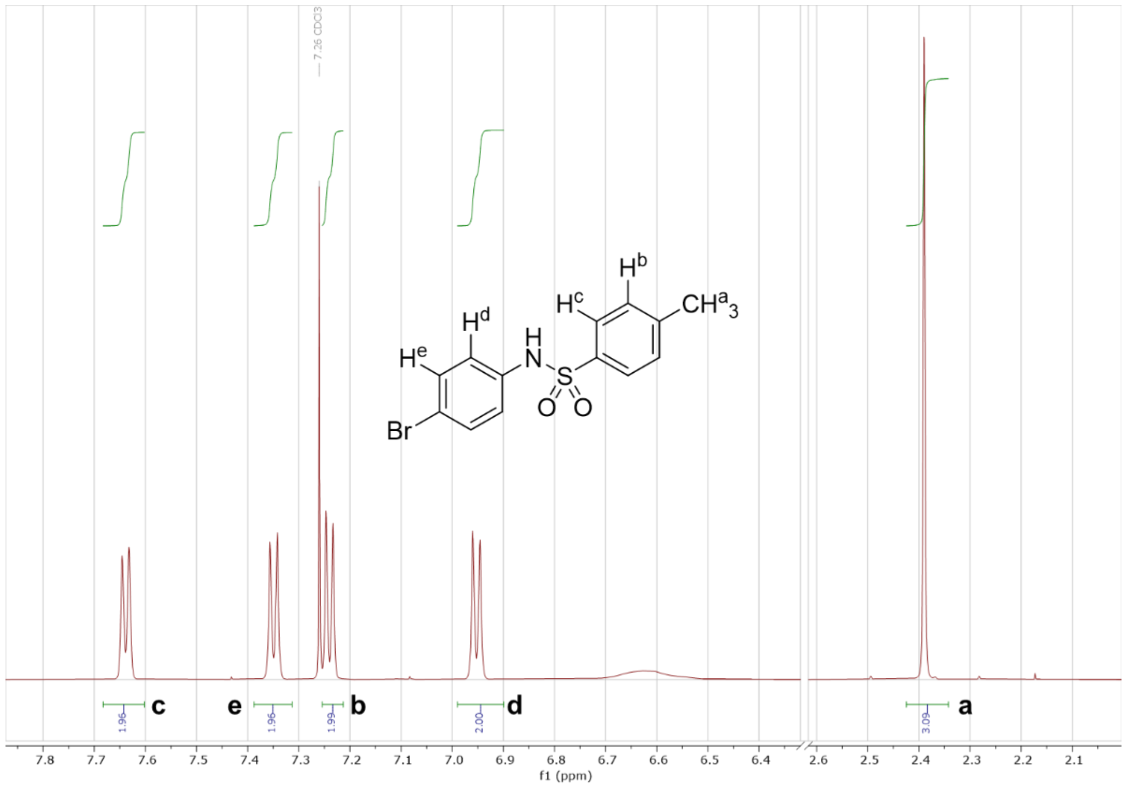

Figure S17 - ^{1}H NMR spectrum of *N*-tosyl-4-bromoaniline, as obtained in the monitored reaction followed by workup.

5. References

1 S. Rohrbach, M. Šiaučiulis, G. Chisholm, P.-A. Pirvan, M. Saleeb, S. H. M. Mehr, E. Trushina, A. I. Leonov, G. Keenan, A. Khan, A. Hammer and L. Cronin, *Science*, 2022, **377**, 172–180.

2 J. Burés, *Angew Chem Int Ed*, 2016, **55**, 16084–16087.
